# Development and preliminary psychometric evaluation of a modified Chinese instrument for self-rated mastery of diabetes exercise knowledge (DEKS-C)

**DOI:** 10.3389/fpubh.2026.1902317

**Published:** 2026-10-20

**Authors:** Lei Mei, Hongying Pan, Kaiyan Zhu, Dan Yin, Huilan Yao, Mingxia Zhu, Yufeng Zhou, Lan Lv, Jing Shao

**Affiliations:** 1 Nursing Department, Sir Run Run Shaw Hospital, Zhejiang University School of Medicine, Hangzhou, China; 2 School of Nursing and Institute of Nursing Research, School of Medicine, Zhejiang University, Hangzhou, China; 3 Department of Nursing, The Fourth Affiliated Hospital of School of Medicine, and International School of Medicine, International Institutes of Medicine, Zhejiang University, Yiwu, China; 4 The D. H. Chen School of Universal Health, Zhejiang University, Hangzhou, China

**Keywords:** cognitive interviewing, cross-cultural adaptation, diabetes mellitus type 2, modified instrument, nursing assessment, reliability and validity, scale validation, self-rated exercise knowledge mastery

## Abstract

**Background:**

Exercise knowledge is seldom evaluated as a standalone construct in China, and no validated instrument exists for self-perceived mastery of exercise knowledge among patients with type 2 diabetes mellitus (T2DM). Adapting the Turkish Diabetes Exercise Knowledge Scale (DEKS) required item consolidation, supplementary items and a revised response format, yielding a modified, DEKS-derived instrument rather than a direct translation.

**Methods:**

This sequential exploratory mixed-methods study was conducted at a tertiary hospital in Hangzhou, China (February–July 2025). In total, 438 patients with T2DM completed the 17-item DEKS-C following forward-backward translation, content validation and three rounds of cognitive interviewing (*n* = 23). Internal consistency and the exploratory factor structure were evaluated using Cronbach’s α and principal axis factoring with oblimin rotation; factor retention followed Horn’s parallel analysis, with the three-factor solution re-examined on a polychoric matrix.

**Results:**

Content validation consolidated nine overlapping items into three and added three new items. Cognitive interviewing then consolidated or relocated four items and merged one into the foot-care item, producing 17 items scored 1–4 (total range 17–68). Cronbach’s α was 0.967 (corrected item-total correlations 0.668–0.837), the Kaiser-Meyer-Olkin measure 0.965, and Bartlett’s test χ^2^(136) = 6945.15, *p* < 0.001. Floor and ceiling effects were 13.47 and 8.90%, arising entirely from the 59 all-lowest-response and 39 all-highest-response respondents, the former answering faster (median 89 vs. 232 s, *p* < 0.001). Horn’s parallel analysis identified three substantially intercorrelated factors (|r| = 0.51–0.79) explaining 55.73–71.23% of total variance (71.23% in the full sample), corresponding to exercise benefits cognition, exercise implementation and exercise safety; an equivalent structure emerged on a polychoric matrix. One pattern coefficient nevertheless exceeded unity and inter-factor correlations were elevated, leaving the solution provisional.

**Conclusion:**

The DEKS-C showed high internal consistency and a three-factor grouping that was broadly stable across sensitivity samples and alternative correlation matrices, supporting content relevance. However, because the factors were substantially intercorrelated and the structure remains unverified, the proposed multidimensional model cannot be regarded as firmly established; independent multicenter and multiregional confirmatory factor analysis is required.

## 1 Introduction

The 11th edition of the International Diabetes Federation (IDF) Diabetes Atlas estimates that 589 million adults aged 20–79 years were living with diabetes globally in 2024 (11.1%), with prevalence projected to rise to 853 million by 2050. China bears the world’s largest disease burden, with over 140 million affected individuals (1). Lifestyle modification interventions can reduces diabetes incidence and slow disease progression (2–4).

Physical exercise, a core cornerstone of lifestyle modification strategies, lowers diabetes incidence (5), diminishes the risk of diabetes-related complications and enhances patients’ quality of life (6). As glycaemic control is heavily reliant on patient treatment adherence (7), robust validated assessment tools are required to evaluate exercise-related knowledge and identify knowledge gaps that impede exercise-related adherence.

Well-validated tools including the Diabetes Knowledge Questionnaire and Diabetes Knowledge Test assess broad-ranging general diabetes knowledge yet incorporate only a small number of exercise-focused items (8), By contrast, physical-activity questionnaires such as the International Physical Activity Questionnaire (IPAQ) evaluate general physical activity, rather than diabetes-specific exercise-related needs, barriers and safety considerations (9). Existing Chinese exercise instruments are predominantly knowledge-attitude-practice instruments that contain relatively few items specifically targeting exercise-knowledge items (8, 10, 11). A narrative literature search across PubMed, Web of Science, CNKI and Wanfang databases, covering publications up to December 2025 yielded no dedicated, validated scale for assessing self-rated mastery of exercise knowledge among Chinese patients living with T2DM. Notably, as this was only a narrative rather than a formal systematic review, this outcome solely represents the results of this targeted search and does not constitute a formally established evidence gap.

The Diabetes Exercise Knowledge Scale (DEKS), originally developed by Ercan in 2022, evaluates exercise-related knowledge among Turkish individuals living with diabetes and demonstrates satisfactory psychometric performance (12). Several items exhibited semantic overlap, and its original five-point response scale posed comprehension challenges for older participants with lower educational attainment. Accordingly, the cross-cultural adaptation process encompassed item consolidation, the addition of safety-focused items, and simplification of the response-option format. Given that these changes altered the item set, response scale and overall score range, the resultant instrument is a DEKS-derived modified and DEKS-derived instead of a linguistically equivalent direct Chinese translation. No claims are made regarding structural equivalence or cross-national score comparability. Consequently, it captures self-rated mastery of the knowledge content described within each individual item, rather than objectively measured factual knowledge; it is therefore not designed to function as an objective knowledge test.

Cognitive interviewing is a qualitative technique used to explore how participants interpret scale items, enabling researchers to identifies ambiguous, confusing or culturally incongruous survey content (13–15); Since literal translation cannot ensure that patients across diverse educational backgrounds interpret items as intended, cognitive interviews were implemented prior to quantitative testing. This study had four primary objectives: (1) to translate and culturally adapt the original DEKS to generate a modified Chinese instrument (DEKS-C) with documented item consolidation and addition; (2) to refine item wording via cognitive interviewing; (3) to assess content validity, internal consistency and exploratory factor structure among Chinese patients living with T2DM; and (4) to investigate how robust the derived structural findings are against extreme and uniform response patterns.

## 2 Methods

### 2.1 Study design

This sequential exploratory mixed-methods study consisted of a qualitative phase (cross-cultural translation and cognitive interviewing), succeeded by a quantitative psychometric-evaluation phase (psychometric evaluation), conducted at Sir Run Run Shaw Hospital, Zhejiang University School of Medicine, a tertiary hospital in Hangzhou, China. Formal written permission for cross-cultural adaptation of the DEKS was secured from its original developer. This study received ethical approval from the hospital ethics committee (approval no. 2024–2,663-01), and all enrolled participants provided written informed consent. Revisions generated from cognitive interviews were incorporated into the version used in the quantitative phase. Thus, qualitative findings directly shaped the item pool and response-option format that underwent subsequent psychometric assessment.

Patients living with T2DM were recruited via convenience sampling from inpatient wards within the endocrinology department of the same hospital between February and July 2025. Participants completed the DEKS-C electronically via a mobile-survey platform during their hospital admission. Participants either self-administered the questionnaire on a tablet or smartphone. For participants who preferred support or experienced reading difficulties, a trained research nurse administered the scale: each item was read aloud, and participant responses were recorded verbatim, with no paraphrasing or explanatory elaboration of item content permitted. All submitted questionnaire were reviewed item by item for completeness and logical consistency, while the survey platform automatically recorded questionnaire completion times (Section 2.6). Supervised questionnaire administration and record-by-record review explain the absence of missing item responses, even though 46.8% of participants had only received primary-school-level education or less.

### 2.2 Scale translation

The DEKS is a self-report questionnaire that assesses general exercise-related knowledge without reference to a fixed time frame (12). Its four dimensions and 25 items—health benefits (6 items), exercise program (9 items), foot care during exercise (4 items) and general precautions (6)—are scored on a 5-point Likert scale (0 = strongly disagree to 4 = strongly agree) and converted to a 0–100 total score, with higher scores indicating greater exercise knowledge. The full instrument is provided in Supplementary Table S5. In the original study (*n* = 279) the content validity index was 0.79, the KMO value was 0.891, four factors explained 71.8% of the variance and Cronbach’s α was 0.943.

The study adhered to the Cognitive Interviewing Reporting Framework (CIRF) checklist (16) and the COSMIN (Consensus-based Standards for the Selection of Health Status Measurement Instruments) standards for evaluating the quality of content validity studies of patient-reported outcome measures (17, 18). The DEKS-C was developed in accordance with the COSMIN criteria (19–21) and the 2024 practical guide for translation, cross-cultural adaptation, and verification of measurement tools (22), covering forward translation, synthesis, back-translation, expert review and content validation, pretesting and submission to the original author (23–27). Two independent bilingual translators, both native Chinese speakers (one an endocrinologist and the other an English teacher without a medical background), produced simplified Chinese versions C1 and C2. A third researcher then compared these versions with the original English DEKS for ambiguities and discrepancies in vocabulary, sentence structure, and meaning. These discrepancies were resolved through joint discussion involving the two forward translators and the third researcher; any remaining disagreements were referred to the expert committee. Two further bilingual researchers (native Chinese speakers with extensive overseas experience, neither previously exposed to the original DEKS) independently back-translated the draft into English (E1 and E2). An expert committee then invited experts in diabetes nursing education, nursing management or clinical endocrinology to compare E1 and E2 with the original scale, semantically inconsistent items being integrated into a comprehensive back-translated version (E3). Substantial modifications were approved in writing by the original developer.

The original five-point agreement format was not retained, because cognitive interviews showed that agreement anchors did not fit the construct: DEKS-C items state facts about exercise in diabetes rather than opinions. The task was understood as a judgment of the extent to which participants had mastered the content of each item. Participants also found fine-grained numeric ratings difficult to use and could not reliably map partial knowledge onto an intermediate value. The response format was therefore finalized as a 4-point labeled self-rated mastery scale (1 = completely unaware to 4 = completely clear and already practicing), with the neutral midpoint removed and an instruction added stating that there are no right or wrong answers. Because the response scale differs from that of the source instrument, the DEKS-C must be evaluated using its own measurement evidence (20). The total score is obtained by summing the 17 items (and ranges from 17 to 68).

Construct and intended use. Each DEKS-C item states a fact about exercise in diabetes and asks respondents to what extent they already know and practice that content. The instrument therefore captures self-rated mastery of exercise knowledge rather than objectively verified knowledge, and it is distinct from measures of exercise self-efficacy, which assess perceived capability rather than knowledge content. Three consequences follow: the score is self-referential and cannot substitute for a knowledge test; second, the instrument is intended for descriptive and educational use—characterizing the content areas about which a patient feels least certain—and no diagnostic, screening or risk cut-off is proposed; third, the highest response option combines knowing with practicing, so at the upper end of the scale knowledge and behavior cannot be separated. How closely these self-ratings correspond to objectively verified knowledge has not been examined, therefore, convergent validity against an objective measure of exercise knowledge remains to be established.

### 2.3 Qualitative cognitive interviewing

Cognitive interviews were undertaken to refine Version 2 of the DEKS-C (the 22-item pre-interview version; Figure 1; Supplementary Table S4). Inclusion criteria comprised age ≥ 18 years, T2DM diagnosed according to American Diabetes Association criteria (28), and the capacity to read simplified Chinese. Exclusion criteria included severe diabetes-related complications, mental illness or cognitive impairment affecting independent completion, and language or hearing impairment. All interviews were delivered face-to-face within a quiet hospital room by a trained researcher experienced in qualitative research and diabetes care. Each session lasted 20–60 min and was audio-recorded for subsequent verbatim transcription. A semi-structured interview guide was used to gather participant feedback regarding general comprehension, scale instructions, item wording and response-option selections (Table 1).

**Figure 1:**
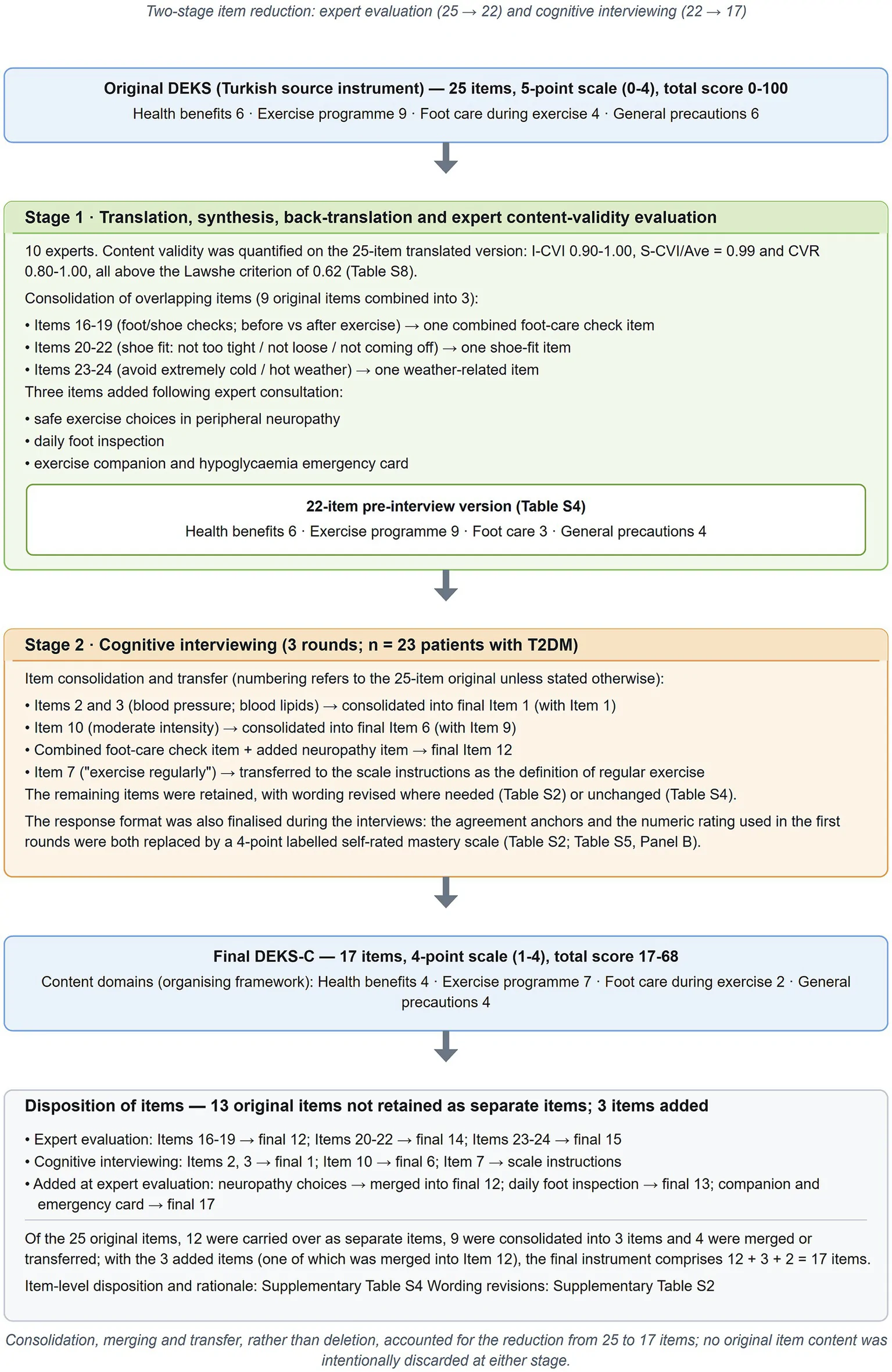
Development and item-reduction process of the DEKS-C, from the original 25-item DEKS to the final 17-item DEKS-C, showing the stage and rationale of each item-revision decision.

**Table 1:** Cognitive interview outline.

Sections	Interview questions
1. Basic information	Age, sex, diabetes duration, educational attainment, marital status, occupation. Daily exercise habits: frequency, type, and duration.
2. Item comprehension & feedback	Please explain the meaning of this item in your own words. Are there any words or phrases you find confusing or difficult to understand? What does the term“regular exercise” mean to you? Does this match your real-life circumstances? Do you consider this item important for exercise-related guidance for people living with diabetes? If yes or no, please explain why. Do you think this item contains any ambiguous wording? Do you have any suggestions to revise this item for clarity?
3. Cultural & linguistic appropriateness	Are the examples (e.g., Tai Chi, square dancing) close to your daily life? Are there any locally popular forms of physical activity that ought to be added or modified within the scale?
4. Scale structure & scoring	Is the grouping of items (e.g., health benefits, foot care) reasonable? Is the scoring instruction easy to understand? Is the scale comprehensive enough to cover key exercise knowledge for diabetes? Do you need to add or delete any items? How long does it take you to complete the scale? Do you feel fatigued? Are there any difficult-to-answer items? Why?
5. Open-ended questions	What are your overall impressions, strengths, and weaknesses of the scale? Did you encounter any difficulties while completing this scale? If so, please elaborate. What further information or guidance would you like the scale to provide?

Three successive rounds of cognitive interviews were performed, whereby each participant took part in one round. Recruitment for each round proceeded until thematic data saturation was achieved (29–33). Following each interview the researcher transcribed and analyzed the text within 24 h (34), extracting feedback pertaining to individual items, before marking, merging and categorizing duplicate content. Any identified ambiguities were cross-checked against the respective interviewee. The expert panel subsequently determined whether an item should be revised or deleted: items were deleted when comprehension barriers persisted despite rewording or when content duplicated other items in the same dimension. Problematic items were evaluated against the four-stage cognitive response process (comprehension, retrieval, judgment and response), and revisions anchored each item to its targeted measurement construct.

### 2.4 Quantitative validation of DEKS-C among Chinese T2DM patients

Across the 17-item scale, the participant-to-item ratio reached 25.8:1 based on 438 valid returned questionnaires, This value exceeded the recommended ratio of 7–10 participants per item as well as the minimum sample size of 200 participants required for structural-validity and exploratory-factor-analysis studies (20, 35); no formal power calculation was performed because the study was exploratory. Eligibility criteria mirrored those applied during those of the cognitive interviews, however interviewees were not eligible, and participants who withdrew consent or returned questionnaires with at least 10% missing item responses were predefined for exclusion; no participant fulfilled either exclusion criterion, and all 438 questionnaires were retained for analysis.

### 2.5 Psychometric evaluation

Content validity was evaluated by a 10-member expert panel comprising physicians and nurses with at least a bachelor’s degree and more than 5 years’ experience in diabetes, endocrinology or metabolic disorders (Supplementary Table S3). Experts rated every item independently, with no group-level discussion and no visibility of other panelists’ ratings. Item relevance was scored on a four-point ordinal scale (1 = not relevant to 4 = highly relevant), and a free-text field was provided for suggested item revisions. Following Polit and Beck (36), ratings of 3 or 4 were categorized as relevant. This identical dichotomisation rule was also applied when calculating the content validity ratio (CVR), whereby an expert rating of 3 or 4 was counted as “essential” (ne). Accordingly, The I-CVI (item-level content validity index) and the CVR therefore shared a single cut-point (36, 37). The I-CVI corresponded to the proportion of panelists rating an item 3 or 4 and the S-CVI/Ave the mean I-CVI, values of at least 0.78 and 0.90, respectively, being regarded as acceptable; CVR = (ne - N/2)/(N/2), with *N* = 10 and the critical value is 0.62 (38). Two completed expert forms each lacked a single item rating (items 15 and 22); these were treated as missing rather than imputed, this approach avoids artificial inflation of the resulting CVR estimates the CVR. Two important methodological points require explicit clarification. First, the panel made a single relevance judgment per item. Therefore, the indices reported herein dichotomise these ratings and do not adopt Lawshe’s original three-category rating framework (essential, useful but not essential, not necessary). Consequently, this calculation constitutes a secondary, transparently derived check instead of an independent source of evidence, being a monotonic function of the same proportion underlying the I-CVI. Second, the I-CVI and the S-CVI/Ave were prioritized as the primary sources of the primary content-validity evidence. This approach aligns with published recommendations advising against using CVR as the main metric for evaluating patient-reported outcome measures (36, 37).

Statistical analyses were performed using SPSS 26.0 (IBM Corp., Armonk, NY, USA) and R 4.4.3, utilizing the psych 2.5.6 and GPArotation 2025.3–1 packages. Descriptive statistics (frequency, percentage, mean ± standard deviation) described participants’ characteristics, and item-level distributions were summarized as the number and percentage selecting each of the four response options, alongside calculations of skewness and kurtosis values. Internal consistency was assessed using Cronbach’s α and McDonald’s ω (39), and item analysis relied on corrected item-total correlations (CITCs). Cronbach’s α with each item deleted was additionally computed for the 17-item total scale. The exploratory factor structure was examined using principal axis factoring (PAF) with oblimin rotation. Dataset suitability for factor analysis was verified via the Kaiser-Meyer-Olkin (KMO) measure and Bartlett’s test of sphericity. Factor retention decisions were guided by Horn’s parallel analysis (40) (psych:fa.parallel(), principal-axis extraction, 1,000 resampled datasets, 95th-percentile criterion). Observed eigenvalues alongside their corresponding 95th-percentile reference values were exported from this parallel-analysis routine (Table 2, Panel A). One- to four-factor PAF solutions were also examined, given that the original DEKS comprised four theoretical dimensions four dimensions; the analysis was repeated in each sensitivity sample, and the three-factor PAF was fitted on a polychoric correlation matrix and compared with the Pearson-based solution. A salient loading was defined *a priori* as |λ| ≥ 0.40, cross-loading required a secondary loading also reaching |λ| ≥ 0.40. Item-to-factor allocation was performed according to each item’s highest-magnitude loading coefficient. Floor and ceiling-effect magnitudes below 15% were regarded as negligible (21). The investigation is reported in compliance with the COSMIN standards for studies on measurement properties (17, 20) and, for the cross-sectional component, with the Strengthening the Reporting of Observational Studies in Epidemiology (STROBE) statement (41).

**Table 2:** Factor retention and comparison of one- to four-factor PAF solutions (*n* = 438).

Sample	N	Factors retained by parallel analysis
**Panel A. Factor retention**		
Full sample (S0)	438	3
Sensitivity sample S1	335	3
Sensitivity sample S2	340	3
Sensitivity sample S3	379	3
Sensitivity sample S4	328	3

Factors retained	Extraction sums of squared loadings	Cumulative variance explained (%)	Variance added (%)	Retention decision
Panel B. Comparison of one-to four-factor principal-axis factoring (PAF) solutions.				
1	10.783	63.43	63.43	Not retained
2	10.826, 0.711	67.86	4.43	Not retained; parallel analysis indicated three factors
3	10.861, 0.727, 0.521	71.23	3.37	Retained
4	10.879, 0.744, 0.526, 0.264	73.02	1.79	Not retained; two-item factor

Horn’s parallel analysis was implemented via psych:fa.parallel() using principal-axis factor extraction (fa = “fa,” fm = “pa”), 1,000 resampled datasets and the 95th-percentile retention criterion (quant = 0.95) in R 4.4.3 (psych 2.5.6). Observed eigenvalues for factors 1–4 and their 95th-percentile reference values were, respectively: S0 10.783, 0.593, 0.405 and 0.162 versus 0.569, 0.354, 0.289 and 0.239; S1 7.713, 0.837, 0.608 and 0.237 versus 0.635, 0.407, 0.331 and 0.273; S2 7.727, 0.837, 0.608 and 0.236 versus 0.626, 0.404, 0.326 and 0.268; S3 8.942, 0.728, 0.532 and 0.203 versus 0.599, 0.377, 0.311 and 0.256; S4 7.499, 0.860, 0.625 and 0.241 versus 0.660, 0.407, 0.340 and 0.274. In every sample the first three observed eigenvalues exceeded their reference values and the fourth did not, the difference at the fourth eigenvalue being −0.077 (S0), −0.037 (S1), −0.032 (S2), −0.053 (S3) and −0.033 (S4). These eigenvalues derive from the SMC-reduced correlation matrix, whereas Panel B reports the sums of squared loadings of the factors actually extracted; the two align for the one-factor solution because that factor explains essentially all of the common variance of the reduced matrix, and diverge whenever further latent factors are extracted additional factors are extracted. Panel B presents the one- to four-factor principal-axis solutions obtained from the reduced correlation matrix applying the SPSS default extraction convergence criterion (0.001). Eigenvalues correspond to extraction sums of squared loadings and cumulative variance explained is their sum divided by the number of items (17). Extraction converged after 4, 10, 9 and 10 iterations for the one-, two-, three- and four-factor solutions, respectively, and the three-factor solution matches the SPSS footnote of nine iterations. The four-factor solution yielded a two-item factor and was therefore not treated as a confirmed structure. Because the factors are correlated, rotated sums of squared loadings cannot be summed to compute the total variance explained. PAF, principal axis factoring.

### 2.6 Response-pattern and data-quality checks

Given that the DEKS-C uses a unidirectional four-point format without reverse-scored items, three response patterns were defined *a priori*: floor and ceiling effects, the proportions scoring the theoretical minimum (17) or maximum (68) (21); uniform responding, zero within-person variance; and maximum run length of consecutive identical responses. The platform recorded completion time for every questionnaire as an objective indicator of possible inattentive responding, Completion-time metrics and participant characteristics among uniform-response participants were compared against those of non-uniform respondents (*n* = 335) via the Mann–Whitney U-test and χ^2^ test (Supplementary Table S7).

Primary analyses were conducted on the full dataset comprising the complete sample of 438 respondents, no respondent being excluded. Four predefined sensitivity analyses examined whether the structural conclusions depended on these anomalous response patterns: (S1) excluding all respondents with zero within-person variance (*n* = 335); (S2) excluding only uniform extreme responders (*n* = 340); (S3) excluding only all-minimum responders (*n* = 379); and (S4) excluding respondents with a longest string ≥ 15 (*n* = 328). In each sensitivity subsample, Cronbach’s α, CITCs, KMO, Bartlett’s test, Horn’s parallel analysis, the cumulative variance explained and the factor correlations were recomputed (Supplementary Table S6).

## 3 Results

### 3.1 Development of DEKS-C

The DEKS-C preserves the four content domains of the original scale as its organizing framework and consists of 17 items: health benefits (4), exercise program (7), foot care during exercise (2) and general precautions (4). The item wording and response options are provided in Supplementary Table S5 and the development is summarized in Figure 1 and Supplementary Table S4.

### 3.2 Expert-panel assessment of content validity

The expert panel for content-validity assessment comprised 10 panelists. Their demographic and professional profiles are detailed in Supplementary Table S3.

#### 3.2.1 Content validity

This expert panel assessed content validity for the initial translated 25-item version of the DEKS-C. Item-level content validity indices reached 1.00 for 23 items and 0.90 for the remaining two (items 15 and 22), yielding an S-CVI/Ave of 0.99. Content validity ratios spanned 0.80 to 1.00 and all surpassing the Lawshe critical value of 0.62 for a 10-member panel: all 10 experts assigned a rating of 23 items 3 or 4 (CVR = 1.00), and 9 of 10 did so for items 15 and 22 (CVR = 0.80). For these two items one rating was left blank on each of two expert submission forms (items 15 and 22). Given that a missing rating is not an endorsement, ne = 9 for both items and no value was imputed, yielding an I-CVI of 9/10 = 0.90. The member who left item 22 blank also annotated it as overlapping with item 21. Item-level values for all 25 items are given in Supplementary Table S8. No item fell below the criterion, so none was deleted. Item consolidation decisions were grounded in semantic overlap and convergent evidence gathered from cognitive interviews (Figure 1). Nine semantically overlapping items were consolidated into three revised items, while three supplementary items were introduced. This process produced the 22-item pre-interview prototype (Figure 1; Supplementary Table S4). These indices therefore apply exclusively to the 25-item translated version: the content validity of the final 17-item DEKS-C was not re-quantified and relies upon the panel’s assessment of semantic overlap, the cognitive interview evidence and panel consensus (Section 4.4).

### 3.3 Cognitive interview findings and scale revision

The 23 cognitive-interview participants had a mean age of 52.17 ± 14.04 years; 14 (60.87%) were male. Thirteen took part in round 1 (P1–P13; 51.54 ± 16.74 years), five in round 2 (P14–P18; 52 ± 9.19 years) and five in round 3 (P19–P23; 54 ± 12.39 years). The mean interview duration was 30 min (Table 3; Supplementary Table S1).

**Table 3:** Characteristics of patients involved in cognitive interview.

Characteristics	Mean ± standard deviation or n (%)
Age (years)	52.17 ± 14.04
Sex	
Male	14 (60.87%)
Female	9 (39.13%)
Educational attainment	
Primary school	6 (26.09%)
Junior high	7 (30.43%)
High school	5 (21.74%)
College	3 (13.04%)
Bachelor’s degree	2 (8.70%)
Occupation	
Farmer	5 (21.74%)
Retired	4 (17.39%)
Clerk	5 (21.74%)
Business owner	3 (13.04%)
Freelancer	3 (13.04%)
Worker	1 (4.35%)
Accountant	1 (4.35%)
Student	1 (4.35%)
Diabetes duration	
≤ 3 months	7 (30.43%)
4 months–3 years	6 (26.09%)
4–10 years	4 (17.39%)
> 10 years	6 (26.09%)

During the first round, most respondents found the items concise and clear; nine reported ambiguous instructions or items, which the expert group revised. These revisions (Supplementary Table S2) predominantly involved wording adjustments that incorporated explanatory text and contextual examples for participants with low educational attainment (Table 4); rather than simplifying the underlying constructs, they restored conceptual equivalence by identifying the stage of the cognitive response process at which difficulty arose and adding targeted clarifying information. Some of that text was embedded directly within the item stems (for example items 6 and 13; Supplementary Table S5); although this improved comprehension, those items may therefore inform respondents rather than measure them.

**Table 4:** Representative item-level revisions implemented following the first round of cognitive interviews.

Original item (pre-interview version)	Problem description	Revision explanation	Revised item
19. Patients with diabetes should wear suitable footwear (including shoes that is unlikely to slip off) when undertaking physical exercise	Insufficient practical operational guidance: detailed instructions are needed for specific procedures of foot examination, criteria for suitable footwear.	Add explanations to the item to make it practical	19. Patients with diabetes should wear breathable, well-fitting shoes with soft soles, which are neither restrictive nor prone to slipping off for exercise, and avoid wearing slippers or high-heeled shoes.
20. Patients with diabetes should avoid physical exercise under conditions of extreme hot or cold ambient temperatures	Participants demonstrated limited comprehension regarding “avoiding exercise in cold/hot weather”	Embed concrete contextual recommendations within the item stem: For summer: “Choose to exercise in the early morning or evening, and avoid high temperature at noon”; For winter: “wear adequate warm clothing and avoid walking on icy roads.”	20. For patients with diabetes, they can choose to exercise in the early morning or evening in summer to avoid high temperature at noon, and need to wear warm clothing in winter to avoid walking on icy roads.

Item numbers are those of the 22-item pre-interview version utilized during cognitive-interview sessions; at that stage original DEKS items 20–22 had been consolidated into item 19 and items 23–24 into item 20 (Supplementary Table S4). Selected illustrative examples; the revision history of the items retained with wording adjustments is provided in Supplementary Table S2, and the disposition of every original item in Supplementary Table S4.

The second round was administered to the revised questionnaire with five respondents (P14–P18); two raised feedback concerning on two items, which the panel reviewed and revised. Across rounds, several older participants with lower educational attainment struggled to match partial knowledge states to which intermediate value on the numeric mastery scale corresponded to partial knowledge, or defaulted to a mid-range value whenever uncertainty occurred, so each round’s review recommended labeled tick options (Supplementary Table S2). In the third round no new comprehension problems emerged and the response format was finalized as the 4-point labeled mastery scale.

During the interviews four additional original items were consolidated or transferred by expert-group decision, on converging evidence from the panel’s assessment of semantic overlap and the interview findings. Items 2 and 3 were merged to form final item 1 (the surviving item of originals 1–3), item 10 into final item 6, and item 7 moved into the introductory scale instructions. The first supplementary newly-added item was combined with the combined foot-care item to generate final item 12. Five items therefore left the pool, reducing the 22-item pre-interview version to the final 17 items.

### 3.4 Quantitative validation of DEKS-C in Chinese T2DM patients

All 438 patients with T2DM completed the 17-item DEKS-C and all questionnaires were retained; characteristics are shown in Table 5, and item-level scores and response-option distributions in Table 6. Skewness spanned −0.48 to 0.35 and kurtosis from −1.66 to −1.38, signaling flat, broadly bimodal distributions; the intermediate options 2 and 3 had the lowest endorsement rates (11.4–18.5% and 16.2–25.3%), whereas option 1 was chosen by 24.2–43.4% and option 4 by 24.9–43.4% of respondents (Table 6).

**Table 5:** Characteristics of patients involved in quantitative validation phase.

Characteristics	n	%
Sex		
Male	222	50.68
Female	216	49.32
Age groups, years		
18–24	2	0.46
25–30	11	2.51
31–35	22	5.02
36–40	77	17.58
41–45	75	17.12
46–50	112	25.57
≥51	139	31.73
Educational attainment		
Primary school and lower	205	46.80
Junior high school	95	21.69
High school/technical secondary school	65	14.84
Junior college/ bachelor’s degree	69	15.57
Master’s degree and above	4	0.91
Marital status		
Unmarried	46	10.50
Married	366	83.56
Divorced	8	1.83
Others	18	4.11
Exercise habit		
No exercise	131	29.91
Occasional exercise	154	35.16
Regular exercise (1–2 times/week)	103	23.52
Frequent exercise (≥3 times/week)	50	11.42

**Table 6:** Item-level scores and response-option distributions for the modified DEKS-derived Chinese-language instrument (DEKS-C), *n* = 438.

Item	Mean ± standard deviation	Option 1, n (%)	Option 2, n (%)	Option 3, n (%)	Option 4, n (%)
Total scale score	42.65 ± 16.95	–	–	–	–
Item 1	2.83 ± 1.22	106 (24.2)	51 (11.6)	91 (20.8)	190 (43.4)
Item 2	2.56 ± 1.23	139 (31.7)	53 (12.1)	106 (24.2)	140 (32.0)
Item 3	2.47 ± 1.24	155 (35.4)	50 (11.4)	105 (24.0)	128 (29.2)
Item 4	2.55 ± 1.22	137 (31.3)	57 (13.0)	111 (25.3)	133 (30.4)
Item 5	2.70 ± 1.23	119 (27.2)	56 (12.8)	100 (22.8)	163 (37.2)
Item 6	2.44 ± 1.22	146 (33.3)	78 (17.8)	91 (20.8)	123 (28.1)
Item 7	2.40 ± 1.21	149 (34.0)	81 (18.5)	91 (20.8)	117 (26.7)
Item 8	2.40 ± 1.23	156 (35.6)	73 (16.7)	85 (19.4)	124 (28.3)
Item 9	2.35 ± 1.24	170 (38.8)	60 (13.7)	91 (20.8)	117 (26.7)
Item 10	2.29 ± 1.24	177 (40.4)	71 (16.2)	76 (17.4)	114 (26.0)
Item 11	2.23 ± 1.24	190 (43.4)	68 (15.5)	71 (16.2)	109 (24.9)
Item 12	2.44 ± 1.26	162 (37.0)	54 (12.3)	89 (20.3)	133 (30.4)
Item 13	2.57 ± 1.25	140 (32.0)	54 (12.3)	98 (22.4)	146 (33.3)
Item 14	2.71 ± 1.25	124 (28.3)	54 (12.3)	87 (19.9)	173 (39.5)
Item 15	2.56 ± 1.22	135 (30.8)	57 (13.0)	111 (25.3)	135 (30.8)
Item 16	2.53 ± 1.24	141 (32.2)	64 (14.6)	91 (20.8)	142 (32.4)
Item 17	2.61 ± 1.21	124 (28.3)	70 (16.0)	99 (22.6)	145 (33.1)

*n* = 438. Option 1 = completely unaware of the content; option 2 = have heard of it, but do not know the content; option 3 = have heard of it, but know only part of the content; option 4 = completely clear about the content and already putting it into practice (full wording provided in Supplementary Table S5, Panel B). Values are numbers and column percentages of respondents; percentages may not sum to 100 because of rounding.

#### 3.4.1 Item analysis

Corrected item-total correlations (CITCs) spanned 0.668 to 0.837 every coefficient surpassed the pre-defined 0.30 retention threshold. Accordingly, no items were removed, and all 17 items proceeded to structural-validity analysis (Table 7).

**Table 7:** Item-level internal consistency of the 17-item DEKS-C (*n* = 438).

Item	Corrected item–total correlation^a^	Cronbach’s α if item deleted
1	0.760	0.965
2	0.795	0.965
3	0.801	0.965
4	0.820	0.964
5	0.668	0.967
6	0.799	0.965
7	0.794	0.965
8	0.802	0.965
9	0.783	0.965
10	0.762	0.965
11	0.798	0.965
12	0.786	0.965
13	0.788	0.965
14	0.721	0.966
15	0.791	0.965
16	0.837	0.964
17	0.780	0.965
Total scale (17 items)	0.668–0.837	0.967

a Correlation between the item and the sum of the remaining 16 items. The right-hand column reports Cronbach’s α for the 16-item scale obtained after deleting that item; no single item, if deleted, would increase the coefficient of the total scale (0.967). McDonald’s ω for the total scale, derived from the one-factor solution, was 0.967; Given that this solution assumes unidimensionality, this coefficient is numerically equivalent to Cronbach’s α and does not provide independent validity evidence. Item numbers correspond to those of the final 17-item DEKS-C (Supplementary Table S5). The three-factor pattern-loading matrix together with item communalities are reported in Table 8.

#### 3.4.2 Internal consistency

Cronbach’s α coefficient for the 17-item full 17-item scale was 0.967, and McDonald’s ω was 0.967. No item deletion increased the coefficient (α if item deleted 0.964–0.967), so none was removed based on reliability-related considerations (Table 7). Given the three retained factors were strongly intercorrelated, internal consistency is reported for the total scale only; in the four sensitivity analyses α was 0.929–0.949.

#### 3.4.3 Structural validity

The dataset of 438 valid participant responses satisfied prerequisites for factor-analytic modeling factor analysis (KMO = 0.965; Bartlett’s test χ^2^(136) = 6945.15, *p* < 0.001). From Horn’s parallel-analysis output the first three observed eigenvalues exceeded their 95th-percentile reference values whereas the fourth did not (observed 10.783, 0.593, 0.405 and 0.162 against reference thresholds of 0.569, 0.354, 0.289 and 0.239). A three-factor structure was indicated in the full sample and in all four sensitivity-analysis subsamples (Table 2, Panel A; Supplementary Table S6). Three factors were therefore extracted and rotated obliquely to form the primary factor-analytic model, while one- to four-factor solutions were compared as theory-driven alternatives (Table 2, Panel B).

The three-factor solution accounted for 71.23% of the total variance within the full dataset, whereby a single dominant factor was evident prior to rotation before rotation. In the rotated pattern matrix (Table 8), items 1–4 loaded primarily on exercise benefits cognition (0.56–0.94), items 7–11 on exercise implementation (0.64–0.95) with item 16 also loading there (0.51), and items 5, 12, 13, 14, 15 and 17 on exercise safety (0.43–0.94). Items 5 and 16 therefore deviated from their theoretical domains, item 6 yielded low, diffuse pattern coefficients (0.38 and 0.32), met the salience criterion on neither factor and was left unassigned, and item 17’s secondary loading of 0.38 was below the salience cut-off. The three extracted factors exhibited substantial inter-factor correlations (|r| = 0.79 between exercise-benefit cognition and exercise implementation; 0.68 between exercise implementation and exercise-safety; 0.71 between exercise-benefit cognition and exercise-safety). Corresponding inter-factor correlations across sensitivity-analysis subsamples ranged 0.51–0.73, and correlation signs are mathematically arbitrary for this oblique-rotation solution. When mapped against the four pre-defined content domains of the original DEKS scale, the exercise-benefit cognition factor corresponded to the original health-benefits domain (items 1–4). The exercise-implementation factor aligned with the original exercise-program domain alongside item 16 (originally belonging to the general-precautions domain; items 7–11 and 16). The exercise-safety factor encompassed items 5, 12–15 and item 17, which were originally sourced from the exercise-program, foot-care and general-precautions domains, respectively. All 17 scale items were retained for final reporting (Table 2). Given the substantial inter-factors were substantially intercorrelated, the pattern coefficients do not sum to the item communalities and a communality can exceed the square of the highest pattern coefficient (item 6); the structure matrix is available within in the supplementary analysis files.

**Table 8:** PAF–oblimin factor loading matrix of the 17-item DEKS-C (three-factor solution, *n* = 438).

Item	Exercise implementation	Exercise safety	Exercise benefits cognition	Communality
1	−0.020	0.323	0.562	0.649
2	−0.054	0.015	0.939	0.824
3	0.234	−0.098	0.758	0.784
4	0.023	0.009	0.893	0.843
5	0.276	0.434	0.048	0.481
6	0.382	0.317	0.196	0.658
7	0.636	0.056	0.184	0.692
8	0.700	0.105	0.083	0.712
9	0.950	−0.069	−0.018	0.792
10	0.688	0.118	0.036	0.646
11	0.805	−0.018	0.093	0.754
12	0.286	0.482	0.123	0.659
13	0.103	0.643	0.163	0.716
14	−0.121	0.942	0.047	0.802
15	0.230	0.604	0.073	0.701
16	0.511	0.308	0.116	0.730
17	0.377	0.524	−0.015	0.667

*n =* 438. Extraction method: principal axis factoring (PAF); rotation: oblimin with Kaiser normalization. Loadings ≥ |0.40| may be interpreted as salient; values are displayed to three decimal places and cross-loadings are retained owing to the exploratory nature of this factor-analytic procedure. Because the factors were substantially intercorrelated (|r| = 0.68–0.79), pattern coefficients do not sum to the communalities, so a communality can exceed the squared value of an item’s highest pattern coefficient (item 6); the corresponding structure matrix is available within the supplementary analysis files. Correlation-factor signs are mathematically arbitrary for oblique-rotation factor-analytic solutions.

#### 3.4.4 Floor and ceiling

Floor and ceiling effects, the proportions achieving the theoretical minimum total score the theoretical minimum (17) or maximum (68), reached 13.47 and 8.90% in the full sample, both below the 15% threshold recommended by Terwee et al. (21). A total of 17 can only occur when participants select the lowest option on every item and 68 requires selection of the highest-ranked option for all items. Accordingly, both floor- and ceiling-effect proportions dropped to 0% after excluding participants exhibiting uniform responses across all 17 items (sensitivity samples S1-S4). Nevertheless, these two proportion alone can not be interpreted as conclusive evidence that the total score is entirely free of extreme-response bias.

### 3.5 Response patterns and data quality

All 438 participants completed every one of the 17 scale items, yielding zero missing item-level responses. Fifty-nine participants (13.47%) selected the lowest ranked response across all 17 items, 39 (8.90%) the highest, and a further 5 (1.14%) option 3 throughout; 103 participants (23.52%) exhibited zero within-person response variance, while 110 (25.11%) produced a maximum run length of ≥ 15 identical consecutive responses. Given that the DEKS-C contains no reverse-scored items, a total score of 17 corresponds to to selecting the lowest ranked response for every scale items. Accordingly, floor-and-ceiling effects and uniform extreme responding inherently coincide by scale design. The remaining 335 respondents (76.5%) altered their selected response option at least once across the 17 items; they are referred to below as non-uniform responders and act as the comparison group for this entire section (Supplementary Table S7; these 335 respondents correspond to sensitivity sample S1 in Section 2.6).

Questionnaire completion times differed substantially between the two extreme subgroups (Supplementary Table S7). Participants who consistently who consistently selected the lowest-ranked option completed the instrument in a median of 89 s (mean 106.8 s), and 72.9% finished within 120 s, compared with a median of 232 s (mean 340.2 s) among the 335 non-uniform responders (Mann–Whitney U, *p* < 0.001; Cohen’s d = −0.77). They lowest-response subgroup was also over-represented among older respondents (83.1% vs. 52.5% were aged 46 years or older; χ^2^(2) = 27.18, *p* < 0.001. Analyses utilized collapsed age categories, because some expected cell counts were below five). Furthermore, over-representation was observed for participants with primary-school-level education or less (66.1% vs. 43.9%; χ^2^(1) = 9.94, *p* = 0.002). By contrast, participants who consistently selected the highest option throughout took a median of 233 s (mean 351.1 s), which did not differ from the non-uniform responders (*p* = 0.78; Cohen’s d = 0.03).

The two extreme-response patterns represent distinct phenomena rather than one unified behavioral profile. The low-end pattern may reflect a composite of limited exercise knowledge, poorer health literacy and rapid straight-line responding, although the present dataset cannot fully disentangle these contributing factors. The high-end pattern showed no behavioral evidence of inattentive responding, but cannot be read as a genuine ceiling of knowledge either, because the endorsed option stated that the respondent was already practicing every item’s content. Furthermore, 48.7% of all-maximum responders reported taking no exercise at all, compared with 33.1% of the non-uniform responders, and only 20.5% exercised regularly or frequently, compared with 37.9% of the non-uniform responders (Supplementary Table S7). A comparable dissociation was also observable for the low-end subgroup, none of the all-minimum responder reported taking no exercise, although 74.6% reported occasional exercise. Collectively, these two response patterns therefore suggest that the response options were not interpreted uniformly. Consequently, neither end of the scale can be read as a purely substantive result.

Findings from all predefined sensitivity analyses are summarized in Supplementary Table S6. Cronbach’s α values spanned 0.929 to 0.967, KMO statistics ranged between 0.933–0.965, Bartlett’s test remained significant throughout (*p* < 0.001) and no CITC fell below 0.30. Mean inter-item correlation stood at 0.633 within the full dataset and varied from 0.435 to 0.521 across sensitivity-analysis subsamples. Horn’s parallel analysis identified a three-factor structure in all five samples, with identical item-to-factor assignments, cumulative variance explained of 55.73–71.23% and absolute factor correlations of 0.51–0.79. The structure was also reproduced on a polychoric correlation matrix, the more appropriate basis for four-point ordinal items. The same three factors and the same grouping of items 1–4, 7–11 and 12–15 were obtained in all five samples, explained 81.26% of the variance in the full sample, with inter-factor correlations spanning a wider range than the Pearson solution (0.47–0.85 versus 0.51–0.79). Item assignment diverged only for the three items closest to the salience threshold: item 5 remained on exercise safety (0.42), item 6 shifted onto exercise implementation (0.45, having met the criterion on no factor in the Pearson solution) and item 17 loaded almost equally on exercise implementation (0.48) and exercise safety (0.48). Item 9 yielded a pattern coefficient of 1.02 in the full sample (0.96–1.00 in the sensitivity samples), which can occur in an oblique solution when the factors are strongly correlated (in this instance r = 0.85) and does not by itself indicate a Heywood case, its communality being 0.885. The overall three-factor grouping therefore survived the sensitivity analyses and the following the switch to polychoric correlations coefficient, but not unchanged in its finer details: three items moved across the salience threshold, one pattern coefficient exceeded unity, and the inter-factor correlations were higher on the polychoric than on the Pearson matrix. Coupled with the strong intercorrelation and the dominance of the first factor in every sample, this suggest that the solution should be regarded as provisional and that the dimensions are closely related facets rather than independent subscales.

## 4 Discussion

### 4.1 Key findings

In the present study, we culturally adapted the original DEKS into Chinese, producing a modified, DEKS-derived instrument, the DEKS-C, through documented item consolidation, item addition and response-format simplification. Content relevance was quantified at the 25-item translated stage (S-CVI/Ave = 0.99); because the item set was then changed, that evidence cannot be generalized to to the final 17-item instrument, whose content was not re-quantified. In 438 patients with T2DM the final 17-item DEKS-C demonstrated high internal-consistency reliability high internal consistency (Cronbach’s α = 0.967, although this estimate is inflated by uniform responding, so the sensitivity estimates of 0.929–0.949 are the more conservative basis for interpretation). Horn’s parallel analysis indicated three factors separating exercise benefits cognition, exercise implementation and exercise safety. Nevertheless, the factors were substantially intercorrelated and the first factor dominated the common variance, so this is not a confirmed multidimensional structure and does not establish structural equivalence or score comparability with the original DEKS.

The structural findings require careful interpretative caution: the three factors correspond only partly to the four content domains of the original DEKS, and two qualifications temper this overall conclusion. First, the first factor accounted for 63.43% of the variance in the one-factor solution and the three factors correlated at |r| = 0.68–0.79. Such large correlation magnitudes cast doubt over the discriminant validity among these latent dimensions. Accordingly, the existence of a strong general dimension therefore remains plausible, and the total score may be the more defensible summary for the underlying construct. Second, several items failed to load onto the factor predicted by their pre-specified theoretical domain: item 5, assigned to the exercise-program domain, loaded on exercise safety (0.43); item 16, assigned to general precautions, loaded on exercise implementation (0.51); item 6 met the salience criterion on no factor; and item 17 showed a borderline secondary loading. The exploratory factor analysis (EFA) is therefore preliminary structural evidence rather than confirmation of a specific latent model, and the divergence from the original DEKS’s four dimensions may reflect the high conceptual relatedness of exercise-knowledge items, the reduced item set or sample-specific response patterns.

Findings derived from response-pattern analyses further qualify how the total scale score ought to be interpreted. Although the floor and ceiling proportions (13.47 and 8.90%) both fell below the 15% threshold, they arose entirely from participants who selected the same option on all 17 items and vanish when those respondents are excluded. Participants in the all-minimum-response subgroup completed the scale substantially more rapidly than non-uniform responders. This pattern is consistent with a mixture of limited knowledge and careless straight-line responding, whereas the all-maximum subgroup showed no such timing signal but reported taking no exercise more often than the non-uniform responders (48.7% vs. 33.1%; *p* = 0.118), discordant with the option they endorsed. Exclusion of uniform responders did not alter the substantive conclusions: three factors were retained in every sensitivity sample with identical item-to-factor assignments, albeit with internal consistency and sampling adequacy were lower than in the full sample (Cronbach’s α = 0.929–0.949 and KMO = 0.933, compared with 0.967 and 0.965). Factor differentiation nonetheless remained limited, particularly in the polychoric solution; the upper end of self-rated mastery is poorly differentiated, and the dissociations at both extremes indicate that the response format itself needs to be revisited, for example by separating knowing from practicing and by adding more difficult items.

### 4.2 Contributions and comparison with previous studies

Exercise assessment tools for T2DM in China are predominantly locally developed knowledge-attitude-practice (KAP)-based composite scales combining exercise knowledge, beliefs and behavior; Liu et al. (10), for example, developed a 37-item scale comprising knowledge (15 items), beliefs (8 items) and behavior (14 items), and the widely adopted Diabetes Knowledge Questionnaire constitutes a general diabetes knowledge measure rather than an exercise-specific tool (8). Knowledge thus tends to be assessed as one component of a broader construct rather than as a distinct measurement target. Against this background, the present study focused specifically on self-rated mastery of this knowledge component, adapting an existing Turkish exercise-knowledge instrument for Chinese patients with T2DM through documented item consolidation and addition. Furthermore, the DEKS-C covers core content domains relevant to safe diabetes-related exercise: exercise principles, indications and contraindications, hypoglycaemia risk, alongside exercise type, intensity and timing. Its development workflow incorporated forward and back translation, expert committee review, three iterative rounds of cognitive-interviewing, and psychometric evaluation based on a comparatively large participant sample (*n* = 438). The literature search outlined above was narrative rather than systematic, identified no dedicated validated Chinese instrument for self-rated mastery of exercise knowledge in T2DM. This outcome solely represents results from this targeted narrative search and cannot be interpreted as definitive proof that no such instrument exists. The present study does not establish that use of the DEKS-C improves outcomes.

Lastly, the DEKS-C differs from the original DEKS in its item set (17 vs. 25 items), response format (four- vs. five-point) and score range (17–68 vs. 0–100). Moreover, the original four-domain latent structure could not be replicated in the present dataset. Accordingly, it constitutes a modified, DEKS-derived instrument rather than a Chinese version of the DEKS. In line with COSMIN guidance, each version is evaluated on its own measurement evidence, and evidence for the original DEKS cannot be extrapolated to the DEKS-C (20). This article therefore reports only the properties the present data support—content validity, internal consistency and exploratory structural validity.

### 4.3 Clinical implications

The DEKS-C may prove valuable in nursing practice and research for assessing patients’ self-rated mastery of exercise-related knowledge. It can help identify knowledge domains where participants report low-confidence levels and which may therefore constitute targets for tailored patient education. No cut-off for individual classification is proposed, so the score should be interpreted in a purely descriptive capacity rather than to triage or diagnose patients, and these applications remain preliminary because the study evaluated measurement properties rather than intervention effectiveness.

### 4.4 Limitations

The present investigation has several limitations. First, convenience sampling from a single tertiary hospital may limit generalizability, and the four-point response format prevents direct score comparability with the original DEKS. Second, the structure was explored without independent cross-validation: no candidate model is confirmed and the high inter-factor correlations (|r| = 0.68–0.79) mean that discriminant validity remains unestablished. A competing account cannot be ruled out, because the factors may partition items by content or difficulty rather than by latent dimension. Items 1–4 had the highest means and items 7–11 the lowest, and a highly homogeneous item set with a dominant first factor and a mean inter-item correlation of 0.633 may likewise produce this type of clustered factor solution such clusters. Discriminating between the two accounts would require comparing the one-, three- and four-factor solutions under a maximum likelihood estimator. For four-point ordinal rating-scale items, modeling should additionally employ the robust weighted-least-squares mean- and variance-adjusted (WLSMV) estimator. The retained factor structure retained by confirmatory factor analysis in an independent sample (Section 4.5). The fourth eigenvalue fell below its 95th-percentile reference in every sample, but by a modest margin in the smaller sensitivity samples (0.032 in S2), and the four-factor solution generated a two-item factor and this model was therefore not selected. Third, internal homogeneity was very high (α = 0.967 and McDonald’s ω = 0.967; KMO = 0.965; mean inter-item correlation 0.633), which points towards partial item redundancy and means that α should be interpreted primarily as an index of a measure of homogeneity rather than as standalone validity evidence of validity. Fourth, 23.52% of participants answered uniformly and were retained in the primary analysis; because such responses inflate α and the magnitude of the first factor, the sensitivity estimates (α = 0.929–0.949) are the more conservative basis for interpretation. Fifth, the highest response option conflates knowing with practicing, and explanatory text was embedded within the stems of several items (Supplementary Table S5), so the top of the scale mixes knowledge with behavior and some items may inform respondents rather than measure them. Sixth, test–retest reliability was not assessed, and, with no attention-check or reverse-scored items, screening for inattentive survey completion relied on completion time and response patterns alone, so extreme responding could not be statistically disentangled from low knowledge. Seventh, the polychoric solution was less well conditioned and yielded higher inter-factor correlations than the Pearson solution (up to 0.85 compared with 0.79), and one pattern coefficient exceeded unity in the oblique solution, which should be interpreted cautiously because highly correlated factors can produce unstable regression-based coefficients. Taken together, these observations does not support strong separation among the extracted dimensions. Eighth, only content validity, internal consistency and structural validity were completed. Cross-cultural validity, measurement invariance, test–retest reliability, criterion validity, hypothesis-based construct validity and responsiveness remain unexamined, and, because no retest was conducted, neither the standard error of measurement (SEM) nor the minimal detectable change (MDC) could be calculated. Ninth, total scale scores capture self-perceived knowledge-mastery rather than objectively verified factual knowledge. Accordingly, the DEKS-C cannot yet be regarded as a fully validated measurement instrument (17, 20). Tenth, all reported content-validity indices were calculated for the initial 25-item translated prototype. Therefore, content-validity metrics for the final 17-item DEKS-C have not been re-quantified. It currently rests on the expert panel’s assessment of semantic overlap, the cognitive interview evidence and panel consensus, and formal re-evaluation by an independent panel would be needed to establish it on the version actually in use.

### 4.5 Future directions

Future research should prioritize independent-sample confirmatory factor analysis in multicenter and multiregional samples, comparing three prespecified candidates: the three-factor model observed in the present study; a one-factor model, still plausible given the dominance of the first factor; and the theory-prespecified four-factor model of the original DEKS domains. Using the currently available datasets, split-half confirmatory analysis and exploratory structural equation or bifactor modeling would test directly whether a single general factor accounts for most of the common variance, and a revision of the response options that separates knowing from practicing ought to be assessed in a new sample. Future studies should also test measurement invariance across demographic and healthcare settings, examine convergent validity of self-rated mastery against an objective exercise-knowledge measure, and comprises assessment of test–retest evaluation (42) together with the corresponding SEM and MDC estimates, responsiveness in longitudinal studies, and explicit tests of discriminant validity.

## 5 Conclusion

The DEKS-C constitutes a modified, DEKS-derived Chinese instrument designed to capture self-rated mastery of diabetes exercise knowledge. Content validity was quantified at the initial 25-item translated prototype, and the final 17-item instrument showed, in Chinese patients with T2DM, high internal consistency and a three-factor grouping that was broadly stable across sensitivity samples and correlation matrices but not in its fine-grained structural details; all 17 items were retained. Given the factors were substantially intercorrelated and the first factor dominant, the multidimensional structure remains preliminary, and independent multicenter and multiregional studies are needed to validate its dimensional architecture, assess further psychometric measurement properties, and determine whether the DEKS-C is appropriate for wider-scale clinical and research application.

## Data Availability

The raw data supporting the conclusions of this article will be made available by the authors, without undue reservation.
