# Supplementary material for: Development and preliminary psychometric evaluation of a modified Chinese instrument for self-rated mastery of diabetes exercise knowledge (DEKS-C)
Source: Front Public Health. 2026 Oct 20;14:1902317. doi: 10.3389/fpubh.2026.1902317 (PMC13727756; doi:10.3389/fpubh.2026.1902317)
Supplement: Supplementary file 1 [file Table_1.docx]

**Supplementary Materials**

Table S1 Characteristics of patients involved in cognitive interview.

| ID | Sex | Age (years) | Occupation | Education level | Diabetes duration |
| --- | --- | --- | --- | --- | --- |
| 1-P1 | Male | 20 | Student | High school | 1 week |
| 1-P2 | Male | 74 | Retired | Junior high | >10 years |
| 1-P3 | Female | 53 | Clerk | College | 5 years |
| 1-P4 | Female | 54 | Farmer | Junior high | 8 years |
| 1-P5 | Male | 42 | Worker | High school | 1 month |
| 1-P6 | Male | 39 | Freelancer | Primary school | 1 year |
| 1-P7 | Female | 59 | Accountant | High school | 3 months |
| 1-P8 | Male | 74 | Retired | Primary school | 1 year |
| 1-P9 | Male | 74 | Retired | Junior high | >20 years |
| 1-P10 | Male | 48 | Business owner | College | >10 years |
| 1-P11 | Female | 60 | Retired | College | 2 years |
| 1-P12 | Male | 35 | Clerk | Bachelor’s degree | 1 year |
| 1-P13 | Female | 38 | Freelancer | Junior high | 3 years |
| 1-P14 | Male | 50 | Clerk | Junior high | 2 months |
| 1-P15 | Female | 53 | Freelancer | High school | 20 years |
| 1-P16 | Male | 56 | Business owner | Primary school | 4 years |
| 1-P17 | Male | 63 | Farmer | High school | 2 weeks |
| 1-P18 | Male | 38 | Clerk | Junior high | 2 weeks |
| 1-P19 | Male | 34 | Business owner | Bachelor’s degree | 9 years |
| 1-P20 | Male | 60 | Farmer | Junior high | >20 years |
| 1-P21 | Female | 50 | Clerk | Primary school | 1 week |
| 1-P22 | Female | 63 | Farmer | Primary school | >30 years |
| 1-P23 | Female | 63 | Farmer | Primary school | 2 years |

Table S2 Details of item revisions. Item numbers are those of the 22-item pre-interview version used in the cognitive interviews, which do not coincide with the original DEKS item numbers beyond Item 15: in particular, pre-interview Item 18 is the added daily foot-inspection item, not original Item 18. The mapping between the two versions is given in the note to Table S4.

| Original item (pre-interview version) | Problem description | Question frequency | Revision description | Revised item | Cognitive response stage addressed | Rationale for revision |
| --- | --- | --- | --- | --- | --- | --- |
| Items 1-6 | The definition and scoring criteria of “regular exercise” in Items 1–6 were unclear | 9 | After discussion, a clear definition of “regular exercise” was added at the beginning of the scale, incorporating participants’ suggestions. | “Regular exercise” in the scale refers to an exercise pattern of at least 3–5 sessions per week, each lasting more than 30 minutes, reaching moderate or higher intensity (e.g., brisk walking, swimming, cycling), and maintained for at least 3 months, adjustable according to individual health status. | Comprehension | Anchors “regular exercise” to the frequency, intensity and duration criteria already embedded in the items. |
| 4. Regular exercise helps reduce the risk of atherosclerosis in diabetic patients | Participants were confused by the technical term “atherosclerosis,” and some could not understand it because of limited education | 5 | The term “atherosclerosis” was retained and explained parenthetically in the item, in line with participants’ suggestions | 4. Regular exercise helps reduce the risk of atherosclerosis (a condition where fatty substances deposit on the inner wall of arteries, forming plaques and leading to thickening and narrowing of the arterial wall) in diabetic patients | Comprehension | Anchors the technical term to its underlying pathophysiological meaning. |
| 5. Regular exercise helps reduce the risk of cardiac events in diabetic patients; 21. If diabetic patients perform high-intensity exercise without medical advice, the risk of cardiac events will increase | Participants were confused by the technical term “cardiac events” in Items 5 and 21, and some could not understand it because of limited education | 4 | After discussion, examples of “cardiac events” were added to the items. | 5. Regular exercise helps reduce the risk of cardiac events (such as myocardial infarction, angina pectoris, arrhythmia, heart failure, cardiac arrest) in diabetic patients; 21. If diabetic patients perform high-intensity exercise without medical advice, the risk of cardiac events (such as myocardial infarction, angina pectoris, arrhythmia, heart failure, cardiac arrest) will increase | Comprehension | Anchors the term to concrete clinical events for the two items that shared it. |
| 6. Regular exercise helps reduce the risk of cerebral infarction in diabetic patients | Participants were confused by the technical term “cerebral infarction,” and some could not understand it because of limited education | 3 | After discussion, “cerebral infarction” was replaced by “stroke (cerebral vascular occlusion)” with an illustrative explanation | 6. Regular exercise helps reduce the risk of stroke (cerebral vascular occlusion) in diabetic patients | Comprehension | Replaces the technical term with a plainer synonym that preserves its meaning. |
| 9. Diabetic patients can start with low-intensity exercise (such as walking, yoga, stair climbing, Tai Chi, etc.), or choose moderate-intensity exercise (including jogging, brisk walking, swimming, etc.) according to their actual situation | Participants were confused by “low-intensity exercise” and “moderate-intensity exercise,” and some did not fully understand them | 4 | After discussion, definitions of “low-intensity exercise” and “moderate-intensity exercise” were added below the item. | 9. Diabetic patients can start with low-intensity exercise (such as walking, yoga, stair climbing, Tai Chi, etc.), or choose moderate-intensity exercise according to their actual situation (including jogging, brisk walking, swimming, cycling, rope skipping, aerobics, badminton, etc.). Explanation: Low-intensity exercise refers to physical activity with low energy expenditure, where the heart rate is maintained below 50% of the maximum heart rate (maximum heart rate ≈ 220 - age), breathing is stable, and conversation can be easily sustained. Moderate-intensity exercise is physical activity with significantly increased energy expenditure, during which the heart rate reaches 50%-70% of the maximum heart rate, breathing accelerates, short conversations are still possible, and slight sweating occurs. | Comprehension | Anchors the two intensity levels to physiological criteria (heart rate, breathing, sweating). |
| 13. Diabetic patients should perform muscle-strength training 2–4 days a week (such as lifting light-weight objects or using resistance bands for stretching, barbells, dumbbells, or fixed equipment) | Participants were confused by “muscle-strength training,” and some older participants or those with limited education did not fully understand it | 2 | After discussion, an explanation of “muscle-strength training” was added to the item, together with examples from daily life | 13. Diabetic patients should perform muscle-strength training 2–4 days a week (if their physical condition permits, they can lift light-weight objects or use resistance bands for stretching, barbells, dumbbells, fixed equipment, etc., including daily activities such as grocery shopping, carrying water buckets, and doing squats while holding bottles) | Comprehension | Anchors the technical term in everyday activities familiar to older patients. |
| 18. Diabetic patients should check their feet daily. If there is decreased skin temperature, cold extremities, or skin that gradually turns white, or changes from dark red to dark purple, or even black purple, timely attention and treatment should be given to avoid injury during exercise, which may cause ulcers or gangrene. | Participants were confused by the technical terms “ulcer” and “gangrene,” and some could not understand them because of limited education | 3 | In line with participants’ suggestions, explanations of “ulcer” and “gangrene” were added to the item to aid comprehension. | 18. All diabetic patients (regardless of age and the presence of foot changes) should check their feet daily. If there is decreased skin temperature, cold extremities, or skin that gradually turns white, or changes from dark red to dark purple, or even black purple, timely attention and treatment should be given to avoid injury during exercise, which may cause ulcers or gangrene. Explanation: An ulcer refers to an open wound formed on the surface of the skin or mucous membrane due to local tissue injury and necrosis, usually reaching below the dermis layer. Gangrene refers to the necrosis of body tissue due to interruption of blood supply (ischemia) or severe infection, accompanied by tissue putrefaction, color change (such as blackening), and foul odor. | Comprehension | Anchors the two terms to their clinical definitions. |
| Response options of all items (original DEKS agreement format: 0 = strongly disagree to 4 = strongly agree; during the interviews each item was rated on a fine-grained numeric mastery scale) | Two problems were recorded. (i) The agreement anchors did not fit the construct being measured: the items are statements of fact about exercise in diabetes rather than matters of opinion, and throughout the interviews participants understood the task as a judgment of how far they had mastered the content of each item rather than as agreement with a proposition. (ii) Several participants, particularly older patients with lower educational attainment, could not reliably map partial knowledge onto an intermediate value of the numeric rating (for example, distinguishing between 40 and 50 or between 60 and 70 points) and tended to default to a mid-range value when uncertain. | 5 | After the second round of interviews, the expert panel replaced the numeric rating with labeled tick options and finalized the response format as a 4-point labeled self-rated mastery scale (1 = completely unaware of the content; 2 = have heard of it but do not know the content; 3 = have heard of it but know only part of the content; 4 = completely clear about the content and already practicing it). The neutral midpoint was removed and the instruction that there are no right or wrong answers was added. | All items were subsequently scored on the 4-point labeled self-rated mastery scale, using the scoring rules given in Table S5, Panel C. | Response process (response selection) | Re-anchoring the response scale to self-rated mastery aligns the response options with the construct actually assessed, replacing an agreement format that did not fit statements of fact. Replacing the numeric rating with labeled options removes the mapping burden for older respondents with lower educational attainment and encourages definitive judgments rather than mid-range defaulting. The revised 4-point labeled format was used in the third round of interviews, in which no further comprehension or response-selection problems were reported. |

Table S3. Basic information of the content validity expert panel.

| ID | Sex | Age, years | Study field | Position/Title | Education level | Years of service |
| --- | --- | --- | --- | --- | --- | --- |
| 1 | Female | 57 | Diabetes | Chief registered nurse | Bachelor | 39 |
| 2 | Female | 48 | Endocrine | Supervisor nurse | Bachelor | 8 |
| 3 | Female | 45 | Endocrine | Deputy chief nurse | Master | 23 |
| 4 | Female | 41 | Endocrine | Supervisor nurse | Master | 19 |
| 5 | Female | 33 | Endocrine | Supervisor nurse | Master | 10 |
| 6 | Male | 54 | Diabetes | Chief physician | Master | 27 |
| 7 | Female | 52 | Diabetes | Physician | PhD | 28 |
| 8 | Male | 43 | Diabetes | Physician | PhD | 12 |
| 9 | Female | 48 | Diabetes | Deputy chief physician | PhD | 22 |
| 10 | Female | 38 | Metabolic diseases | Deputy chief physician | PhD | 8 |

Table S4. Disposition of original DEKS items during adaptation and item reduction

Final DEKS-C development record: 25 original items → 22-item pre-interview version → 17-item final version

| Original item no. | Dimension | Original item content (25-item DEKS) | Adaptation stage and decision | Final item | Rationale |
| --- | --- | --- | --- | --- | --- |
| 1 | Health benefits | Regular exercise helps to lower blood glucose levels in patients with diabetes. | Retained without consolidation; wording revised during cognitive interviewing (Table S2) | Final Item 1 | Surviving base of the consolidation of Items 1-3; the blood-pressure and lipid content of Items 2 and 3 was merged into it. |
| 2 | Health benefits | Regular exercise helps reduce high blood pressure (hypertension) in people with diabetes. | Cognitive interviewing; consolidated with Items 1 and 3 | Final Item 1 | Content overlapped with Items 1 and 3, which address cardiometabolic benefits of regular exercise. The blood-pressure content was retained within the combined item. |
| 3 | Health benefits | Regular exercise helps reduce harmful blood lipids (bad cholesterol) in people with diabetes. | Cognitive interviewing; consolidated with Items 1 and 2 | Final Item 1 | As for Item 2; the lipid content was retained within the combined item. |
| 4 | Health benefits | Regular exercise helps to reduce the risk of atherosclerosis in patients with diabetes. | Retained without consolidation; wording revised during cognitive interviewing (Table S2) | Final Item 2 | No semantic overlap with other items; the technical term was explained parenthetically rather than replaced. |
| 5 | Health benefits | Regular exercise helps to reduce the risk of cardiac events in patients with diabetes. | Retained without consolidation; wording revised during cognitive interviewing (Table S2) | Final Item 3 | No semantic overlap with other items; examples were added to the technical term. |
| 6 | Health benefits | Regular exercise helps to reduce the risk of cerebral infarction in patients with diabetes. | Retained without consolidation; wording revised during cognitive interviewing (Table S2) | Final Item 4 | No semantic overlap with other items; the technical term was replaced by a plainer synonym. |
| 7 | Exercise program | People with diabetes should exercise regularly. | Cognitive interviewing; transferred to scale instructions | Scale instructions | The statement was generic and did not add distinct knowledge content. The definition of “regular exercise” was therefore moved from the item stem to the scale instructions. |
| 8 | Exercise program | Before exercising, patients with diabetes should undergo a health check to rule out conditions that make exercise unsuitable, including a finger-prick blood glucose test (exercise is not recommended when blood glucose is too high or too low). | Retained without consolidation; wording unchanged | Final Item 5 | No semantic overlap with other items; content central to exercise safety was retained. |
| 9 | Exercise program | Patients with diabetes may start with low-intensity exercise (e.g., walking, yoga, stair climbing, tai chi) and may also choose moderate-intensity exercise (including jogging, brisk walking, swimming, etc.) according to their own circumstances. | Retained without consolidation; wording revised during cognitive interviewing (Table S2) | Final Item 6 | Base of the consolidation with Item 10, which covered continuation of moderate-intensity exercise. |
| 10 | Exercise program | Individuals with diabetes should continue to exercise at moderate intensity. | Cognitive interviewing; consolidated with Item 9 | Final Item 6 | Item 9 already addressed progression from low- to moderate-intensity exercise. Combining the items retained both intensity levels within one item and reduced redundancy. |
| 11 | Exercise program | Before exercising, patients with diabetes should perform a warm-up (targeted stretching to relax the muscles and increase joint flexibility). | Retained without consolidation; wording unchanged | Final Item 7 | No semantic overlap with other items. |
| 12 | Exercise program | Patients with diabetes should perform at least 150 minutes of aerobic exercise per week (e.g., brisk walking, running, square dancing, cycling, swimming, etc.). | Retained without consolidation; wording unchanged | Final Item 8 | No semantic overlap with other items. |
| 13 | Exercise program | Patients with diabetes should perform muscle-strength training on 2-4 days per week (e.g., lifting light objects or using resistance bands for stretching, barbells, dumbbells or fixed equipment). | Retained without consolidation; wording revised during cognitive interviewing (Table S2) | Final Item 9 | No semantic overlap with other items; everyday examples were added to the technical term. |
| 14 | Exercise program | Patients with diabetes may interrupt exercise, with a maximum of 2 consecutive days of rest. | Retained without consolidation; wording unchanged | Final Item 10 | No semantic overlap with other items. |
| 15 | Exercise program | Patients with diabetes should perform balance exercises (e.g., baduanjin, tai chi) on at least 3 days per week to reduce the risk of falls. | Retained without consolidation; wording unchanged | Final Item 11 | No semantic overlap with other items. |
| 16 | Foot care during exercise | Individuals with diabetes-related nerve ending damage (peripheral neuropathy) should check their feet for wounds before exercise. | Expert evaluation; consolidated into an intermediate combined foot-care item | Final Item 12 | Items 16–19 formed a 2 × 2 pattern (before vs after exercise × feet vs shoes/surface). They were combined into one item covering pre- and post-exercise checks of feet, footwear, and surface. |
| 17 | Foot care during exercise | Individuals with diabetes-related nerve ending damage (peripheral neuropathy) should check for stones, nails, etc. in their shoes before exercise. | Expert evaluation; consolidated into an intermediate combined foot-care item | Final Item 12 | Shoe and surface checks were retained within the combined foot-care item. |
| 18 | Foot care during exercise | Individuals with diabetes-related nerve ending damage (peripheral neuropathy) should check their feet for wounds after exercise. | Expert evaluation; consolidated into an intermediate combined foot-care item | Final Item 12 | The post-exercise foot-check content was retained within the combined item. |
| 19 | Foot care during exercise | Individuals with diabetes-related nerve ending damage (peripheral neuropathy) should check for stones, nails, etc. in their shoes after exercise. | Expert evaluation; consolidated into an intermediate combined foot-care item | Final Item 12 | The post-exercise shoe and surface checks were retained within the combined item. |
| 20 | General precautions | Individuals with diabetes should exercise with shoes that are not too tight. | Expert evaluation; consolidated into an intermediate shoe-fit item; wording revised during cognitive interviewing (Table 3) | Final Item 14 | Items 20–22 addressed the same construct (appropriate and secure footwear) with closely related wording and were combined into one item. |
| 21 | General precautions | Individuals with diabetes should exercise with shoes that are not loose. | Expert evaluation; consolidated into an intermediate shoe-fit item; wording revised during cognitive interviewing (Table 3) | Final Item 14 | Content was retained within the combined shoe-fit item. |
| 22 | General precautions | Individuals with diabetes should exercise with shoes that do not come off easily. | Expert evaluation; consolidated into an intermediate shoe-fit item; wording revised during cognitive interviewing (Table 3) | Final Item 14 | Content was retained within the combined shoe-fit item. |
| 23 | General precautions | Individuals with diabetes should avoid exercising in extremely cold weather. | Expert evaluation; consolidated into an intermediate weather item; wording revised during cognitive interviewing (Table 3) | Final Item 15 | Items 23 and 24 addressed complementary seasonal precautions and were combined into one item covering both cold- and hot-weather conditions. |
| 24 | General precautions | Individuals with diabetes should avoid exercising outdoors in extremely hot weather. | Expert evaluation; consolidated into an intermediate weather item; wording revised during cognitive interviewing (Table 3) | Final Item 15 | Content was retained within the combined seasonal-safety item. |
| 25 | General precautions | If patients with diabetes perform high-intensity exercise without medical advice, the risk of cardiac events increases. | Retained without consolidation; wording revised during cognitive interviewing (Table S2) | Final Item 16 | No semantic overlap with other items; retained as a distinct precaution item. |
| Added 1 | Foot care during exercise | Individuals with diabetes-related peripheral neuropathy should avoid prolonged walking, jogging or walking on uneven ground, and may choose low-load exercise (e.g., walking, cycling, rowing, water-based exercise). | Added after expert evaluation; merged during cognitive interviewing | Final Item 12 | The item addressed exercise safety in peripheral neuropathy, a topic not covered by the original items. During cognitive interviewing, it was considered together with the combined foot-check content and was consolidated into Final Item 12. |
| Added 2 | Foot care during exercise | Patients should inspect their feet daily (skin temperature, skin color changes) and manage abnormalities promptly to avoid exercise-related injury, ulceration or gangrene. | Added after expert evaluation; retained as a separate item | Final Item 13 | Daily foot surveillance extends exercise-specific foot checks to everyday self-care. Cognitive interviewing supported its distinctiveness as a separate content area. |
| Added 3 | General precautions | Patients may exercise with companions, carry a hypoglycaemia emergency card and sugar, and keep the exercise session within an appropriate duration. | Added after expert evaluation; retained as a separate item | Final Item 17 | The item addresses hypoglycaemia preparedness and practical safety during exercise, a topic not covered by the original items. |

**Note.** The reduction from the original 25-item DEKS to the 17-item DEKS-C occurred in two stages. At the expert evaluation stage, nine original items were consolidated into three items (Items 16–19, Items 20–22, and Items 23–24) and three new items were added, yielding a 22-item pre-interview version. Items 16–22 of that version are, in order, the combined foot-care check item, the added item on exercise choices in peripheral neuropathy, the added daily foot-inspection item, the combined shoe-fit item, the combined weather item, original Item 25 and the added item on exercise companions and hypoglycemia preparedness; pre-interview Item 18 is therefore not original Item 18. At the cognitive-interviewing stage, four items were consolidated or transferred (Items 2 and 3 into Item 1, Item 10 into Item 6, and Item 7 into the scale instructions), and Added Item 1 was merged with the combined foot-care item into Final Item 12, yielding the 17-item DEKS-C. All 25 original items therefore appear in this table: 13 were merged into or transferred to another item, and 12 were carried over without being merged, becoming Final Items 1–11 and 16, respectively (Item 1 as the surviving base of the consolidation of Items 1-3, and Item 9 as the base of the consolidation with Item 10). Wording revisions made during cognitive interviewing, and the cognitive-interview evidence supporting them, are reported in Table S2; no original content was intentionally discarded.

Table S5. The final 17-item modified Chinese instrument derived from the DEKS (DEKS-C): item content, response options and scoring rules.

Panel A. Item content of the final 17-item DEKS-C

| Item | Chinese item wording (final DEKS-C) | English back-translation |
| --- | --- | --- |
| Health benefits (4 items) | | |
| 1 | Regular physical activity helps lower blood glucose, blood pressure, and blood lipid levels in individuals with diabetes. | Regular exercise helps to lower blood glucose, blood pressure and blood lipid levels in patients with diabetes. |
| 2 | Regular physical activity helps reduce the risk of atherosclerosis (a condition in which fatty deposits build up on the inner walls of arteries, forming plaques that cause the arterial walls to thicken and harden) in individuals with diabetes. | Regular exercise helps to reduce the risk of atherosclerosis (deposition of fatty substances on the inner wall of the arteries, forming plaques that thicken and harden the arterial wall) in patients with diabetes. |
| 3 | Regular physical activity helps reduce the risk of cardiac events (such as myocardial infarction, angina pectoris, arrhythmias, heart failure, cardiac arrest, etc.) in individuals with diabetes mellitus. | Regular exercise helps to reduce the risk of cardiac events (e.g., myocardial infarction, angina pectoris, arrhythmia, heart failure, cardiac arrest) in patients with diabetes. |
| 4 | Regular physical activity helps reduce the risk of stroke (cerebrovascular event) in individuals with diabetes mellitus. | Regular exercise helps to reduce the risk of stroke (cerebral vascular occlusion) in patients with diabetes. |
| Exercise program (7 items) | | |
| 5 | Diabetic patients should rule out any conditions that would contraindicate physical activity, such as when blood glucose levels are below 4 mmol/L. | Before exercising, patients with diabetes should rule out conditions that make exercise unsuitable, such as a blood glucose level below 4 mmol/L. |
| 6 | Individuals with diabetes can start with low-intensity physical activities such as walking, yoga, climbing stairs, or practicing Tai Chi; alternatively, they may choose moderate-intensity exercises—such as jogging, brisk walking, swimming, cycling, jumping rope, aerobics, or playing badminton—depending on their personal circumstances. Explanation: Low-intensity exercise refers to physical activity characterized by low energy expenditure, a heart rate below 50% of the maximum heart rate (maximum heart rate ≈ 220 – age), steady breathing, and the ability to sustain conversation comfortably. Moderate-intensity exercise refers to physical activity in which energy expenditure increases significantly, the heart rate ranges between 50% and 70% of the maximum heart rate, breathing becomes faster, yet one can still engage in short conversations and experience slight sweating. | Patients with diabetes may start with low-intensity exercise (e.g., walking, yoga, stair climbing, tai chi) and may also choose moderate-intensity exercise (e.g., jogging, brisk walking, swimming, cycling, rope skipping, aerobics, badminton) according to their own circumstances. Explanation: low-intensity exercise is physical activity with low energy expenditure, in which the heart rate remains below 50% of the maximum heart rate (maximum heart rate ≈ 220 − age), breathing is steady and conversation can be easily sustained; moderate-intensity exercise is physical activity with a marked increase in energy expenditure, in which the heart rate reaches 50%–70% of the maximum heart rate, breathing accelerates, short conversation is still possible and slight sweating occurs. |
| 7 | Individuals with diabetes can perform targeted stretching or other warm-up exercises before physical activity to relax their muscles and increase joint flexibility. | Before exercising, patients with diabetes may perform a warm-up such as targeted stretching to relax the muscles and increase joint flexibility. |
| 8 | Individuals with diabetes should engage in at least 150 minutes of weekly aerobic exercise per week, such as brisk walking, running, square dancing, cycling, or swimming. | Patients with diabetes should perform at least 150 minutes per week of aerobic exercise such as brisk walking, running, square dancing, cycling or swimming. |
| 9 | Diabetic patients may engage in muscle-strength training 2–4 days per week, provided their physical condition permits; examples include lifting light weights or stretching with resistance bands, as well as using barbells, dumbbells or stationary equipment, such as performing daily tasks like grocery shopping, carrying a bucket of water or doing wall squats. | If their physical condition permits, patients with diabetes may perform muscle-strength training on 2–4 days per week, such as lifting light objects or stretching with resistance bands, barbells, dumbbells or fixed equipment, including everyday activities such as carrying groceries, carrying water buckets and doing wall squats. |
| 10 | Diabetic patients may discontinue exercise for no more than 2 days. | Patients with diabetes may interrupt exercise for no longer than 2 consecutive days. |
| 11 | Diabetic patients should engage in balanced exercise—at least three days per week—such as standing on one foot, walking on tiptoes, practicing the Eight-Section Exercise, or Tai Chi—to reduce the risk of falling. | Patients with diabetes should perform balance exercises such as single-leg standing, walking on tiptoe, baduanjin or tai chi on at least 3 days per week to reduce the risk of falling. |
| Foot care during exercise (2 items) | | |
| 12 | Diabetic patients experiencing numbness or reduced sensation in their hands or feet should inspect their footwear before and after physical activity, check whether the exercise venue contains stones, nails or other hazards, avoid walking on gravelly or uneven surfaces, and refrain from prolonged walking; instead, they may opt for activities such as walking on flat terrain, rowing or water-based exercise. | Patients with diabetes who have numbness or reduced sensation in the hands or feet should check their shoes and inspect the exercise surface for stones, nails and similar objects before and after exercising, avoid walking on gravel or uneven ground and avoid prolonged walking, and may choose activities such as walking on level ground, rowing or water-based exercise. |
| 13 | Diabetic patients should check daily for any wounds or changes in skin color (e.g., whitening or bluing); any abnormality should be addressed promptly to prevent injuries during physical activity that could lead to skin breakdown, infection or even necrotic blackening. Explanation: an ulcer is an open wound formed on the surface of the skin or mucous membranes as a result of local tissue injury or necrosis, typically extending below the dermis; gangrene refers to the necrosis of body tissues due to interrupted blood supply (ischemia) or severe infection, accompanied by tissue decay, color change (e.g., blackening), and foul odor. | Patients with diabetes should check their feet every day for wounds and changes in skin color (whitening or purple discoloration) and deal with any abnormality promptly so as to avoid exercise-related injury that may cause skin breakdown, infection or even necrosis with blackening. Explanation: an ulcer is an open wound on the skin or mucosal surface caused by local tissue injury and necrosis, usually extending below the dermis; gangrene is tissue necrosis caused by interrupted blood supply (ischemia) or severe infection, accompanied by tissue putrefaction, color change (e.g., blackening) and foul odor. |
| General precautions (4 items) | | |
| 14 | Diabetic patients should choose shoes that are breathable, well-fitting, have soft soles, are not tight, and do not easily come off; they should avoid slippers or high-heeled shoes. | Patients with diabetes should choose breathable, well-fitting shoes with soft soles that are neither tight nor easy to slip off, and should avoid slippers and high-heeled shoes. |
| 15 | Diabetic patients may engage in physical activity in the early morning or in the evening during the summer to avoid the high temperatures at noon; in winter, they should wear warm clothing and avoid walking on icy roads. | In summer, patients with diabetes may exercise in the early morning or evening to avoid the high midday temperature; in winter they should wear warm clothing and avoid walking on icy roads. |
| 16 | Diabetic patients who engage in high-intensity physical activities—such as sprinting, vigorous team sports or rapid swimming—without prior medical evaluation face an increased risk of experiencing cardiac events (e.g., myocardial infarction, angina pectoris, arrhythmia, heart failure, cardiac arrest). | If patients with diabetes perform high-intensity exercise such as fast running, vigorous ball games or fast swimming without medical assessment, their risk of cardiac events (e.g., myocardial infarction, angina pectoris, arrhythmia, heart failure, cardiac arrest) increases. |
| 17 | Diabetic patients engaging in physical activity outdoors may consider accompanying themselves with a companion or carrying a hypoglycemia emergency card and sugar-containing foods; the recommended exercise duration is between 30 minutes and 1 hour. | When exercising away from home, patients with diabetes may arrange to exercise with a companion, carry a hypoglycaemia emergency card and sugar-containing food, and keep the exercise session to 30–60 minutes. |

Panel B. Response options of the final DEKS-C

| Score | Chinese response option (final DEKS-C) | English equivalent |
| --- | --- | --- |
| 1 | 完全不知道 | Completely unaware of the content. |
| 2 | 听说过且不清楚内容 | Have heard of it, but do not know the content. |
| 3 | 听过但只清楚部分内容 | Have heard of it, but know only part of the content. |
| 4 | 完全清楚并实践 | Completely clear about the content and already practicing it. |

Panel C. Administrative specifications and scoring rules

| Specification | Detail |
| --- | --- |
| Respondent task | For each of the 17 statements, respondents indicate how well they already know and practice the exercise-related knowledge described in that statement. The instrument therefore records self-rated mastery of the knowledge addressed by each item, not agreement with the statement. |
| Response scale in the original DEKS and in the DEKS-C | The original DEKS uses an agreement-based response scale, on which respondents indicate how far they agree with each statement. The DEKS-C uses the 4-point self-rated mastery scale given in Panel B, on which respondents indicate how well they consider that they already know and practice the knowledge described in each statement. |
| Scale instructions | The questionnaire is introduced as a survey of the respondent’s mastery of diabetes exercise knowledge; respondents are told that there are no right or wrong answers and are asked to respond according to their actual situation. “Regular exercise” is defined in the instructions as an exercise pattern of at least 3–5 sessions per week, each lasting more than 30 minutes (e.g., brisk walking, swimming, cycling), maintained for at least 3 months and adjustable according to individual health status. |
| Scoring | The total score is the untransformed sum of the 17 item scores. All items are scored in the same direction and none is reverse-scored, so that a higher option always denotes a higher level of mastery. |
| Total score range and direction | 17–68; higher total scores indicate a higher level of self-rated mastery of the exercise-related knowledge addressed by the 17 items. |
| Missing data | Questionnaires with ≥10% missing item responses or obvious logical errors were excluded before analysis; no item-level imputation was performed. No missing item responses occurred among the 438 analyzed questionnaires. |
| Interpretation | No diagnostic, screening or risk cut-off values are proposed. The total score is intended to describe the overall level of self-rated mastery of exercise-related knowledge and to help identify content areas that may warrant patient education, not to classify individual patients. Because the responses are self-reported, the score reflects self-rated rather than objectively verified knowledge. |
| Availability and permissions | Permission to translate and adapt the DEKS was obtained from the original developer by email. The DEKS-C is provided for non-commercial research and educational use, with citation of this article and of the original DEKS. The instrument is not intended for commercial use; any further modification, translation or redistribution requires the permission of the original developer. |

Note: Panel A gives the final Chinese item wording administered in the quantitative phase and a back-translation into English prepared for this report; the back-translation is provided for reference and is not a validated English version of the instrument. Item numbers in Panel A correspond to the item numbers used in Tables 5–8 of the main text. Panel B gives the four response options as administered: they form a single ordered continuum from no knowledge to knowledge that is already put into practice, with no neutral mid-point, and respondents rated how well they already knew and practiced the knowledge described in each statement rather than their agreement with it. Panel C summarizes the administrative specifications and scoring rules of the instrument. Original DEKS items were consolidated or relocated rather than deleted; the disposition of all 25 original items is given in Table S4 and its accompanying note, and the revision history of individual items in Table S2.

Table S6. Sensitivity analyses of reliability, sampling adequacy and structural dimensionality of the 17-item DEKS-C in the full sample and in four pre-specified sensitivity samples.

Panel A. Pearson-based analyses in the full sample and in the four pre-specified sensitivity samples

| Indicator | S0 | S1 | S2 | S3 | S4 |
| --- | --- | --- | --- | --- | --- |
| Sample definition | All respondents (primary analysis) | Zero within-person variance excluded | Uniform extreme responders excluded | All-minimum responders excluded | Longest string ≥ 15 excluded |
| n | 438 | 335 | 340 | 379 | 328 |
| Respondents excluded, n (%) | 0 | 103 (23.52) | 98 (22.37) | 59 (13.47) | 110 (25.11) |
| Cronbach’s α (17 items) | 0.967 | 0.933 | 0.933 | 0.949 | 0.929 |
| Corrected item–total correlation range | 0.668–0.837 | 0.429–0.742 | 0.429–0.742 | 0.511–0.779 | 0.407–0.736 |
| Mean inter-item correlation | 0.633 | 0.448 | 0.449 | 0.521 | 0.435 |
| KMO | 0.965 | 0.936 | 0.937 | 0.951 | 0.933 |
| Bartlett’s χ²(136) | 6945.1 | 3246.6 | 3305.4 | 4503.9 | 3070.1 |
| Bartlett’s p | <0.001 | <0.001 | <0.001 | <0.001 | <0.001 |
| Factors suggested by Horn’s parallel analysis | 3 | 3 | 3 | 3 | 3 |
| Cumulative variance explained, three-factor solution (%) | 71.23 | 56.68 | 56.76 | 62.48 | 55.73 |
| Absolute factor correlations (range) | 0.68–0.79 | 0.52–0.66 | 0.52–0.66 | 0.56–0.73 | 0.51–0.65 |
| Factor correlation: implementation–safety | 0.683 | 0.534 | 0.533 | 0.576 | 0.524 |
| Factor correlation: implementation–benefits cognition | 0.793 | 0.658 | 0.658 | 0.727 | 0.646 |
| Factor correlation: safety–benefits cognition | 0.706 | 0.522 | 0.523 | 0.556 | 0.510 |
| Primary item assignment | — (reference sample) | Identical to S0 | Identical to S0 | Identical to S0 | Identical to S0 |

Panel B. Sensitivity analysis based on the polychoric correlation matrix (three-factor PAF with oblimin rotation, all five samples)

| Sample | Absolute factor correlationsᵃ | Primary item assignment |
| --- | --- | --- |
| S0 (n = 438) | 0.71, 0.85, 0.72 | Same as the Pearson-based solution, except that item 17 loaded almost equally on implementation (0.48) and safety (0.48) |
| S1 (n = 335) | 0.50, 0.71, 0.52 | Same as the Pearson-based solution; item 17 loaded on both implementation (0.44) and safety (0.47) |
| S2 (n = 340) | 0.49, 0.71, 0.52 | Same as the Pearson-based solution; item 17 loaded on both implementation (0.44) and safety (0.47) |
| S3 (n = 379) | 0.58, 0.78, 0.58 | Same as the Pearson-based solution, except item 5 (implementation 0.37 vs safety 0.33); item 17 loaded on both implementation (0.50) and safety (0.44) |
| S4 (n = 328) | 0.47, 0.70, 0.50 | Same as the Pearson-based solution; item 17 loaded on both implementation (0.43) and safety (0.47) |

Note: S0 is the primary analysis and includes all 438 respondents; S1–S4 are the four pre-specified sensitivity samples defined by the response-pattern rules described in Section 2.6. Horn’s parallel analysis was performed using psych::fa.parallel() with principal-axis factor extraction (fa = "fa", fm = "pa"), 1,000 resampled datasets and the 95th-percentile criterion (quant = 0.95). Three factors were indicated in all five samples. The same three-factor solution was subsequently examined using principal axis factoring with oblimin rotation. Absolute factor correlations are reported because the signs of obliquely rotated factors are arbitrary.

Table S7. Completion time and characteristics of respondents with uniform response patterns and of respondents with non-uniform response patterns (n = 438).

| Characteristic | All-minimum  responders  (n = 59) | All-maximum  responders  (n = 39) | Non-uniform  respondersᵃ  (n = 335) | P₁ᵇ | P₂ᵇ |
| --- | --- | --- | --- | --- | --- |
| Panel A. Completion time and response behavior | | | | | |
| Completion time, seconds — median (IQR) | 89.0 (65.5–127.0) | 233.0 (136.0–420.5) | 232.0 (132.0–416.5) | < 0.001 | 0.783 |
| Completion time, seconds — mean (SD) | 106.8 (61.4) | 351.1 (328.9) | 340.2 (326.5) |  |  |
| Completed within 120 s, n (%) | 43 (72.9) | 8 (20.5) | 73 (21.8) | < 0.001 | 0.854 |
| Completed within 90 s, n (%) | 30 (50.8) | 4 (10.3) | 40 (11.9) | < 0.001 | 0.757 |
| Cohen’s d for completion timeᶜ | -0.77 | 0.03 | Reference |  |  |
| Panel B. Sociodemographic and exercise characteristics | | | | | |
| Sex, n (%)  χ²(1) |  |  |  | 0.910 | 0.516 |
| Male | 30 (50.8) | 18 (46.2) | 173 (51.6) |  |  |
| Female | 29 (49.2) | 21 (53.8) | 162 (48.4) |  |  |
| Age group, years, n (%)  χ²(6) |  |  |  | < 0.001 | 0.859 |
| 18–24 | 1 (1.7) | 0 (0.0) | 1 (0.3) |  |  |
| 25–30 | 1 (1.7) | 1 (2.6) | 9 (2.7) |  |  |
| 31–35 | 1 (1.7) | 2 (5.1) | 19 (5.7) |  |  |
| 36–40 | 4 (6.8) | 9 (23.1) | 63 (18.8) |  |  |
| 41–45 | 3 (5.1) | 4 (10.3) | 67 (20.0) |  |  |
| 46–50 | 13 (22.0) | 12 (30.8) | 86 (25.7) |  |  |
| ≥51 | 36 (61.0) | 11 (28.2) | 90 (26.9) |  |  |
| Education level, n (%)  χ²(4) |  |  |  | 0.015 | 0.933 |
| Primary school and lower | 39 (66.1) | 18 (46.2) | 147 (43.9) |  |  |
| Junior high school | 12 (20.3) | 7 (17.9) | 73 (21.8) |  |  |
| High school/technical secondary school | 4 (6.8) | 7 (17.9) | 54 (16.1) |  |  |
| Junior college or bachelor’s degree | 4 (6.8) | 7 (17.9) | 57 (17.0) |  |  |
| Master’s degree and above | 0 (0.0) | 0 (0.0) | 4 (1.2) |  |  |
| Marital status, n (%)  χ²(3) |  |  |  | 0.185 | 0.963 |
| Unmarried | 3 (5.1) | 4 (10.3) | 39 (11.6) |  |  |
| Married | 50 (84.7) | 33 (84.6) | 278 (83.0) |  |  |
| Divorced | 1 (1.7) | 1 (2.6) | 6 (1.8) |  |  |
| Others | 5 (8.5) | 1 (2.6) | 12 (3.6) |  |  |
| Exercise habit, n (%)  χ²(3) |  |  |  | < 0.001 | 0.118 |
| No exercise | 0 (0.0) | 19 (48.7) | 111 (33.1) |  |  |
| Occasional exercise | 44 (74.6) | 12 (30.8) | 97 (29.0) |  |  |
| Regular exercise (1–2 times/week) | 13 (22.0) | 4 (10.3) | 83 (24.8) |  |  |
| Frequent exercise (≥3 times/week) | 2 (3.4) | 4 (10.3) | 44 (13.1) |  |  |

Note: Responses were given on the 4-point mastery scale described in Table S5 (Panel B). All-minimum responders selected the lowest option (1 = completely unaware of the content) and all-maximum responders the highest option (4 = completely clear about the content and already practicing it) on all 17 items; non-uniform responders changed response option at least once across the 17 items. Five additional respondents (1.14%) selected option 3 (“heard of it, but know only part of the content”) on all 17 items; they are not shown separately and were not included in the non-uniform comparison group. ᵃReference group for all comparisons. ᵇP₁ compares all-minimum responders with non-uniform responders, and P₂ compares all-maximum responders with non-uniform responders; Mann–Whitney U tests were used for completion time and χ² tests for the categorical variables (the row label gives the test and its degrees of freedom and the cell gives the P value, and percentages are column percentages). ᶜCohen’s d was computed on raw completion times using the pooled standard deviation and is expressed relative to non-uniform responders. Completion time in seconds was recorded automatically by the electronic survey platform for every questionnaire. Age and education categories are those used in Table 4 of the main text. Because the uniform-pattern subgroups are small, some expected cell counts in the χ² tests were below 5 (minimum expected counts: age group 0.30 and 0.10; education level 0.60 and 0.42; marital status 1.05 and 0.73); these P values should therefore be interpreted with caution. Analyses using collapsed categories gave the same conclusions: for all-minimum versus non-uniform responders, age group χ²(2) = 27.18, P < 0.001; education level χ²(1) = 9.94, P 0.002; and exercise habit χ²(2) = 50.89, P < 0.001. For all-maximum versus non-uniform responders no categorical variable differed significantly (all P > 0.05), and all expected cell counts in these collapsed analyses were ≥ 5. Among all-minimum responders, 83.1% were aged 46 years or older (versus 52.5% of non-uniform responders) and 66.1% had completed primary school or lower (versus 43.9%).

Supplementary Analysis Files

The supplementary analysis files contain the numerical output underlying Tables 7, 8, S4, S6 and S7, including the principal-axis factor solutions, factor correlations, item assignments, sensitivity analyses and response-pattern analyses. The SPSS syntax used to reproduce the two- and four-factor solutions and the R syntax used for Horn’s parallel analysis are also provided.

Table S8. Item-level content validity of the original 25-item DEKS-C (10-expert panel; ratings of 3 or 4 treated as essential).

| **Item** | **Dimension** | **N** | **Experts rating 3-4 (ne)** | **I-CVI** | **CVR** | **Meets criterion** |
| --- | --- | --- | --- | --- | --- | --- |
| 1 | Health benefits | 10 | 10 | 1.00 | 1.00 | Yes |
| 2 | Health benefits | 10 | 10 | 1.00 | 1.00 | Yes |
| 3 | Health benefits | 10 | 10 | 1.00 | 1.00 | Yes |
| 4 | Health benefits | 10 | 10 | 1.00 | 1.00 | Yes |
| 5 | Health benefits | 10 | 10 | 1.00 | 1.00 | Yes |
| 6 | Health benefits | 10 | 10 | 1.00 | 1.00 | Yes |
| 7 | Exercise program | 10 | 10 | 1.00 | 1.00 | Yes |
| 8 | Exercise program | 10 | 10 | 1.00 | 1.00 | Yes |
| 9 | Exercise program | 10 | 10 | 1.00 | 1.00 | Yes |
| 10 | Exercise program | 10 | 10 | 1.00 | 1.00 | Yes |
| 11 | Exercise program | 10 | 10 | 1.00 | 1.00 | Yes |
| 12 | Exercise program | 10 | 10 | 1.00 | 1.00 | Yes |
| 13 | Exercise program | 10 | 10 | 1.00 | 1.00 | Yes |
| 14 | Exercise program | 10 | 10 | 1.00 | 1.00 | Yes |
| 15 | Exercise program | 10 | 9 | 0.90 | 0.80 | Yes |
| 16 | Foot care during exercise | 10 | 10 | 1.00 | 1.00 | Yes |
| 17 | Foot care during exercise | 10 | 10 | 1.00 | 1.00 | Yes |
| 18 | Foot care during exercise | 10 | 10 | 1.00 | 1.00 | Yes |
| 19 | Foot care during exercise | 10 | 10 | 1.00 | 1.00 | Yes |
| 20 | General precautions | 10 | 10 | 1.00 | 1.00 | Yes |
| 21 | General precautions | 10 | 10 | 1.00 | 1.00 | Yes |
| 22 | General precautions | 10 | 9 | 0.90 | 0.80 | Yes |
| 23 | General precautions | 10 | 10 | 1.00 | 1.00 | Yes |
| 24 | General precautions | 10 | 10 | 1.00 | 1.00 | Yes |
| 25 | General precautions | 10 | 10 | 1.00 | 1.00 | Yes |

Note. The 10 panel members rated the relevance of each item on a four-point scale (1 = not relevant, 2 = weakly relevant, 3 = fairly relevant, 4 = highly relevant). Following Polit and Beck, ratings of 3 or 4 were considered relevant, and the same dichotomization was used for the content validity ratio, an item rated 3 or 4 being counted as essential (ne). CVR = (ne - N/2)/(N/2) with N = 10 and a critical value of 0.62. One expert left a single rating blank for item 15 and another for item 22 (the latter annotated as overlapping with item 21); missing ratings were not counted as endorsements and were not imputed, giving ne = 9, CVR = 0.80 and I-CVI = 9/10 = 0.90 for those two items. All 25 items exceeded the criterion.
